# RNF2 regulates Wnt/ß-catenin signaling via TCF7L1 destabilization

**DOI:** 10.1038/s41598-023-47111-x

**Published:** 2023-11-13

**Authors:** Youngmu Koo, Wonhee Han, Byeong-Rak Keum, Leila Lutz, Sung Ho Yun, Gun-Hwa Kim, Jin-Kwan Han

**Affiliations:** 1 Department of Life Sciences, Pohang University of Science and Technology, 77 Cheongam-Ro, Nam-Gu, Pohang, Gyeongbuk 37673 Republic of Korea; 2 F. M. Kirby Neurobiology Center, Department of Neurology, Boston Children’s Hospital, Harvard Medical School, Boston, MA 02115 USA; 3 Center for Research Equipment, Korea Basic Science Institute, Cheongju, 28119 Republic of Korea; 4 Research Center for Bioconvergence Analysis, Korea Basic Science Institute, Cheongju, 28119 Republic of Korea

**Keywords:** Molecular biology, Cell signalling

## Abstract

The Wnt signaling pathway is a crucial regulator of various biological processes, such as development and cancer. The downstream transcription factors in this pathway play a vital role in determining the threshold for signaling induction and the length of the response, which vary depending on the biological context. Among the four transcription factors involved in canonical Wnt/ß-catenin signaling, TCF7L1 is known to possess an inhibitory function; however, the underlying regulatory mechanism remains unclear. In this study, we identified the E3 ligase, RNF2, as a novel positive regulator of the Wnt pathway. Here, we demonstrate that RNF2 promotes the degradation of TCF7L1 through its ubiquitination upon activation of Wnt signaling. Loss-of-function studies have shown that RNF2 consistently destabilizes nuclear TCF7L1 and is required for proper Wnt target gene transcription in response to Wnt activation. Furthermore, our results revealed that RNF2 controls the threshold, persistence, and termination of Wnt signaling by regulating TCF7L1. Overall, our study sheds light on the previously unknown degradation mechanism of TCF7L1 by a specific E3 ligase, RNF2, and provides new insights into the variability in cellular responses to Wnt activation.

## Introduction

The Wnt signaling pathway has been extensively studied due to its involvement in various cellular processes, such as proliferation, differentiation, and migration^1^. Dysregulation of this pathway has been linked to the development of various diseases, including cancer^2,3^. The canonical Wnt pathway is initiated by the binding of Wnt ligands to Frizzled receptors and the LRP5/6 coreceptor, which leads to the stabilization and accumulation of β-catenin in the cytoplasm^4^. The stabilized β-catenin then translocates to the nucleus and functions as a transcriptional coactivator by binding to T cell factor (TCF) and lymphoid enhancer factor (LEF) transcription factors^5–8^. Therefore, the transcriptional output of the Wnt signaling pathway is largely influenced by the availability and activity of the TCF/LEF family transcription factors^9^.

Recent studies have shown that each transcription factor may have unique functions, contrary to the previous belief that they mainly serve as complementary factors in a single signaling pathway^10,11^. In particular, TCF7L1, a transcription factor that plays a crucial role in the canonical Wnt signaling pathway, has been found to possess a unique transcriptional repressor activity, which is in contrast to the other TCF/LEF transcription factors^12–20^. TCF7L1 acts as a ‘brake’ in Wnt signaling, which means that when TCF7L1 is highly expressed, the transcription of Wnt target genes can be suppressed even in the presence of induced Wnt signaling. Conversely, when there is a deficiency in TCF7L1, Wnt target genes can be transcribed even without Wnt signaling being induced, due to the presence of basal levels of β-catenin in the nucleus^21^. Therefore, it is important to maintain an appropriate level of TCF7L1, while understanding the regulatory mechanism of TCF7L1 expression is crucial.

Despite extensive research, the exact method of TCF7L1 degradation has so far remained a question mark. In our study, we identify a specific E3 ligase, RNF2, which regulates the expression of TCF7L1. RNF2 continuously degrades TCF7L1 to maintain an appropriate level of expression, while the level of TCF7L1 expression changes in response to changes in RNF2 expression levels. We have experimentally confirmed that even with the same level of Wnt signaling induction, the expression of Wnt target genes and the response to Wnt signaling can vary greatly, depending on the expression level of TCF7L1, as regulated by RNF2.

These findings shed light on the role of TCF7L1 in cell lines, in which it is abundant, and provide crucial information to understanding the regulation of the Wnt signaling pathway in general cell lines, where TCF7L1 is not prominent^22^. The identification of RNF2, as a specific E3 ligase, which regulates TCF7L1 expression creates new avenues for further research to uncover the precise mechanisms in the regulation of the Wnt signaling pathway and the development of new therapeutic strategies for diseases that involve the dysregulation of the Wnt signaling pathway.

## Results

### TCF7L1 is degraded through ubiquitination following the activation of the Wnt signaling pathway

**Figure 1:**
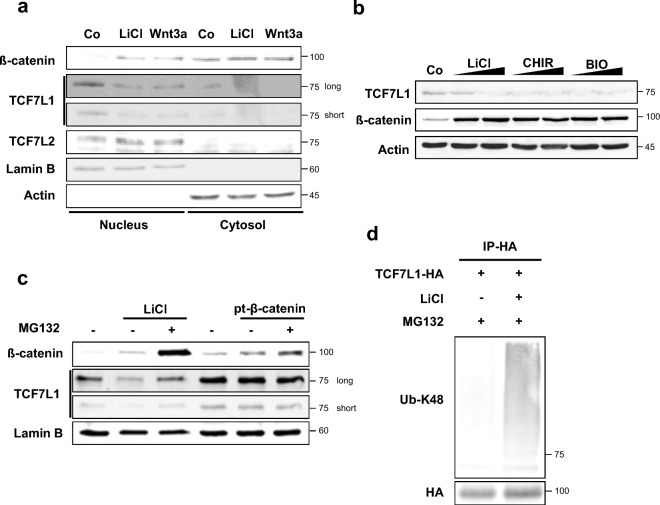
TCF7L1 undergoes degradation through ubiquitination following activation of the Wnt signaling pathway. (**a**) Western blot analysis was performed using HeLa cells. Cells were treated with 50 mM LiCl or mWnt3a conditioned media for 16 h. The cytosolic fraction was lysed with CB buffer, while the nuclear fraction was lysed with RIPA buffer following initial lysis with CB buffer. (**b**) HeLa cells treated with LiCl (25 mM and 50 mM), CHIR99021 (5 μM and 10 μM) and BIO (5 μM and 10 μM) conditioned media for 16 h and lysed with RIPA buffer. (**c**) Western blot analysis was performed using HeLa cells. Cells were treated with LiCl-conditioned media or transfected with the pt-β-catenin plasmid, followed by treatment with 10 μM MG132 for 12 h prior to harvesting. (**d**) In vitro ubiquitination assay was performed using control HeLa cells. TCF7L1-HA (6 μg) was transfected into HeLa cells. Then, the cells were treated with 50 mM LiCl-conditioned media for 16 h, followed by treatment with 10 μM MG132 for 12 h prior to harvesting with IP buffer. The cells were lysed with the IP buffer and subjected to immunoprecipitation with an HA antibody. Ubiquitination levels were determined by anti-UB-K48.

Previous studies have shown that TCF7L1 decreases upon Wnt activation in prominent embryonic stem cells (ESCs) and poorly differentiated breast cancer. To determine whether this mechanism is conserved in non-prominent cells, Wnt signaling was induced in HeLa cells under two conditions: Wnt3a ligand transfection and the inhibition of GSK3b activity by LiCl treatment^23^. Western blot analysis showed that although the expression of TCF7L1 was not strong, it was predominantly expressed in the nucleus and its level specifically decreased upon Wnt activation (Fig. 1a).

When treating with CHIR99021 and BIO, known to inhibit the activity of GSK3b, it was also observed that Wnt signaling was activated through an increase in β-catenin^24,25^. Additionally, a decrease in TCF7L1 was confirmed (Fig. 1b).

Furthermore, to confirm whether the decrease in TCF7L1 by Wnt activation could be rescued by the protease inhibitor MG132, MG132 was added alongside LiCl treatment, which restored the level of TCF7L1 that had previously been decreased by LiCl (Fig. 1c, lane 1, 2, 3). Interestingly, when pt-β-catenin, which is not degraded by the degradation complex due to mutations in its four β-catenin phospho-sites, was transfected to induce Wnt signaling, a decrease in TCF7L1 was not observed (Fig. 1c, lane 4, 5, 6)^26^. This suggests that binding with β-catenin alone is not sufficient to destabilize TCF7L1^27^.

To determine whether the level of ubiquitination of TCF7L1 increases upon Wnt activation by LiCl treatment, a ubiquitination assay was performed. The results showed that the amount of ubiquitinated TCF7L1 increased upon Wnt activation (Fig. 1d). These findings indicate that the mechanism of TCF7L1 degradation upon Wnt activation is relatively conserved and that TCF7L1 is degraded through E3 ligase-mediated ubiquitination.

### RNF2 is a novel positive regulator of the Wnt signaling

**Figure 2:**
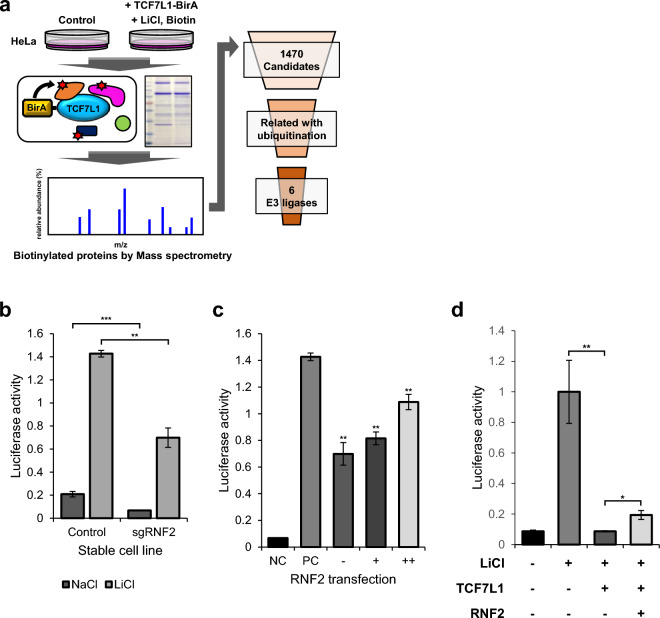
Candidate gene screening through mass spectrometry and luciferase assay. (**a**) Schematic diagram depicting the process of screening candidate genes that interact with TCF7L1. (**b**) TOPflash luciferase assay was performed using control HeLa cells and stable RNF2 knockdown cell lines. Cells were transfected with the indicated plasmids (50 ng TK-Renilla reporter; 200 ng TOPflash) and then treated with 50 mM NaCl and LiCl conditioned media for 16 h. N = 3 independent experiments. (**c**) TOPflash luciferase assay was performed using RNF2 knockdown stable HeLa cells. Cells were transfected with the indicated plasmids (50 ng TK-Renilla reporter; 200 ng TOPflash; 1 μg; 2 μg pCS2 + RNF2) and then treated with 50 mM NaCl and LiCl conditioned media for 16 h. The negative control (Co) was treated with 50 mM NaCl conditioned media. The positive control (PC) is performed using control HeLa cells with 50 mM LiCl conditioned media. N = 3 independent experiments. (**d**) TOPflash luciferase assay was performed using control HeLa cells. Cells were transfected with the indicated plasmids (50 ng TK-Renilla reporter; 200 ng TOPflash; 0.5 μg pCS2 + TCF7L1; 2.5 μg pCS2 + RNF2) and then treated with 50 mM LiCl conditioned media for 16 h. N = 3 independent experiments.

To screen for E3 ligases, HeLa cells were transfected with TCF7L1 and treated with LiCl under BIOID-based mass spectrometry conditions. Subsequently, a total of 1470 candidate genes were identified with the potential to interact with TCF7L1 (Fig. 2a). The list of candidate genes included β-catenin, which is already known to interact with TCF7L1, thus, its inclusion provided some reliability to the list^22^. Through literature study, 15 ubiquitin-related genes were filtered (Table 1), six of which contained E3 ligases (Table 2).

To validate the candidate genes, stable knockdown cells were created using two methods: shRNA and sgRNA. The knockdown efficiency was confirmed using qPCR for shRNA-based knockdown (Fig. S1a), while the sgRNA was verified using a mutation detection kit (Fig. S1b). TCF7L1 is a well-known negative regulator of Wnt, thus, affecting Wnt signaling activity^12–20^. Therefore, luciferase assays were performed using the stable knockdown cells to confirm any changes in the Wnt signaling activity, while any genes that showed significant changes were identified (Fig. S1c,d). Additionally, the changes in endogenous TCF7L1 and the response to Wnt activation from LiCl treatment were confirmed (Fig. S1e).

RNF2 is an E3 ligase that was ranked 39th out of the 1470 genes and expressed a high probability of interacting with TCF7L1. TCF7L1 was confirmed as a negative regulator of Wnt signaling because when cells were transfected with TCF7L1, luciferase activity rapidly decreased in the TOPflash assay^13,21,28^. Therefore, if an E3 ligase induces the degradation of TCF7L1, knockdown of the E3 ligase would result in a relative increase in TCF7L1, thereby resulting in a decrease in Wnt signaling activity. Similarly, RNF2 stable knockdown cells reduced luciferase activity by almost 50%, as expected (Fig. 2b). This decrease was gradually rescued to levels similar to the control (Co) when RNF2 was cotransfected (Fig. 2c). Moreover, transfecting TCF7L1 and overexpressing RNF2 partially restored the decreased luciferase activity, suggesting that the overexpressed RNF2 induced the degradation of TCF7L1 (Fig. 2d).

### RNF2 interacts with TCF7L1 leading to its ubiquitin-mediated degradation

**Figure 3:**
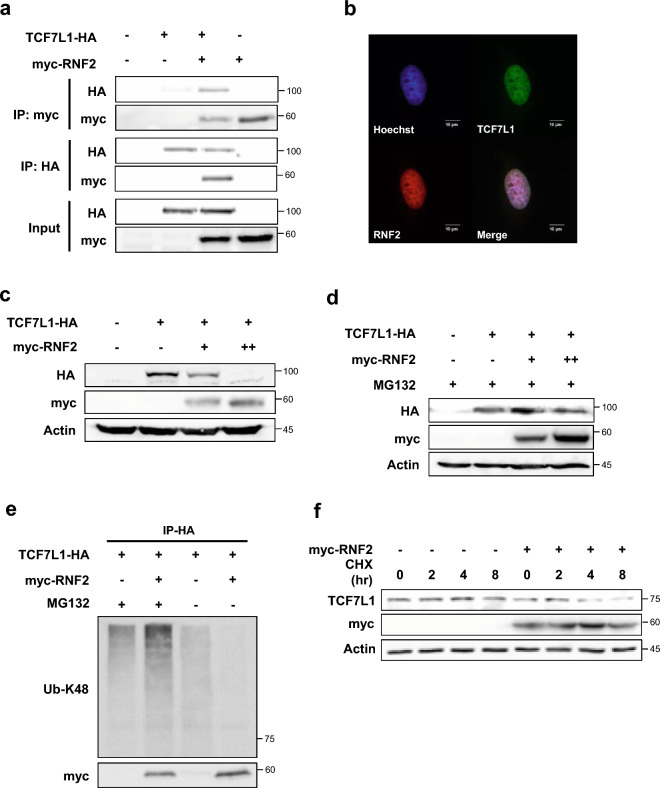
RNF2 interacts with TCF7L1 and destabilizes TCF7L1. (**a**) Coimmunoprecipitation assay was performed using control HeLa cells. The indicated plasmids (6 μg TCF7L1-HA and 6 μg myc-RNF2) were transfected into cells, which were then treated with MG132 (10 μM, 12 h) and subjected to immunoprecipitation using anti-myc and anti-HA antibodies. (**b**) Immunocytochemistry assay was performed using control HeLa cells. Cells were fixed and stained with anti-TCF7L1 (green) and anti-RNF2 (red) after treatment with 10 μM MG132 for 12 h. Scale bar represents 10 μm. (**c**) Western blot analysis was performed using control HeLa cells. TCF7L1-HA (6 μg) and myc-RNF2 plasmids (3 μg and 6 μg) were introduced into cells, which were then lysed with RIPA buffer. (**d**) Western blot analysis was performed using control HeLa cells. TCF7L1-HA (6 μg) and myc-RNF2 plasmids (3 μg and 6 μg) were introduced into cells, followed by treatment with 10 μM MG132 for 12 h before harvest and lysed with RIPA buffer. (**e**) In vitro ubiquitination assay was performed using control HeLa cells. TCF7L1-HA (6 μg) and myc-RNF2 plasmids (6 μg) were introduced into cells, followed by treatment with 10 μM MG132 for 12 h before harvesting. The cells were lysed using the IP buffer and subjected to immunoprecipitation with anti-HA. Ubiquitination levels were determined by anti-UB-K48. (**f**) Cycloheximide chase assay was performed using control 4T1 cells. myc-RNF2 plasmids (6 μg) were introduced into cells, which were then treated with cycloheximide (10 μM) for 2, 4, and 8 h. Cells were lysed with RIPA buffer.

To validate the results of mass spectrometry, coimmunoprecipitation was first conducted to confirm the interaction between TCF7L1 and RNF2 (Fig. 3a). To confirm whether the two proteins colocalized, an immunocytochemistry assay was performed following the treatment with MG132, which confirmed their colocalization in the nucleus (Fig. 3b).

The HeLa and HEK293T cell lines, which were used for validation in this study have low endogenous levels of TCF7L1, which makes it difficult to detect any changes (Fig. S2a). Therefore, to observe changes, exogenous TCF7L1 was transfected, and the changes were observed. Here, TCF7L1 decreased in a dose-dependent manner following co-transfection with RNF2 (Fig. 3c). This decrease was restored when MG132 was added (Fig. 3d). Furthermore, it was observed that overexpression of RNF2 increased the level of ubiquitination (Fig. 3e). However, in the absence of MG132, there was a tendency for the ubiquitination level to decrease, indicating that the TCF7L1 in the lysate had already been degraded following RNF2 overexpression (Fig. S3a).

In the poorly differentiated breast cancer cell line, 4T1, which prominently expresses TCF7L1, a cycloheximide assay was performed, which confirmed that the overexpression of RNF2 decreased the stability of TCF7L1 (Fig. 3f).

### Knockdown of RNF2 stabilizes nuclear TCF7L1

**Figure 4:**
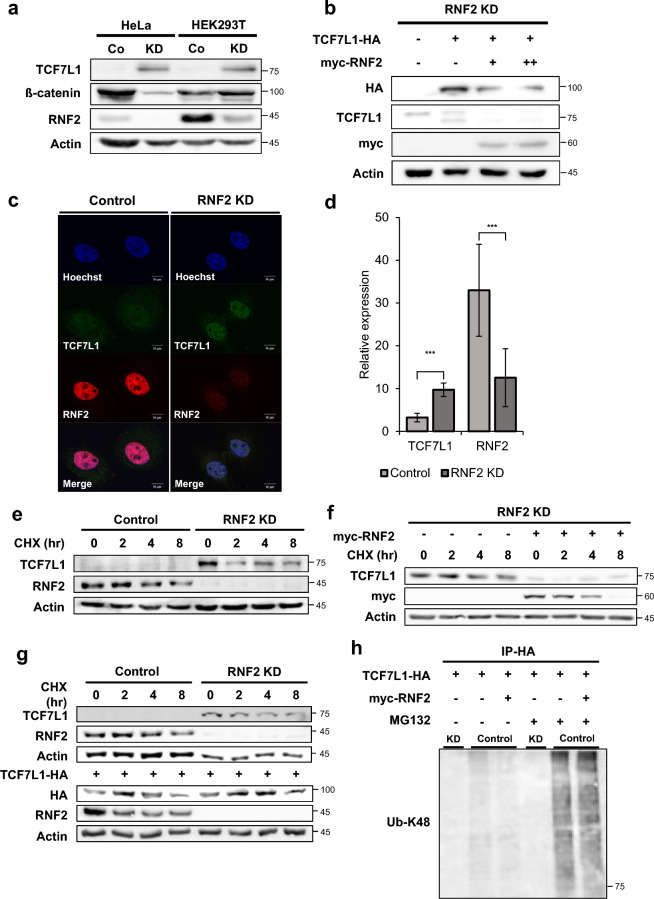
Loss of RNF2 stabilizes nuclear TCF7L1. (**a**) Western blot analysis of control HeLa and HEK293T cells and RNF2 knockdown stable HeLa and HEK293T cells. Endogenous TCF7L1, β-catenin, and RNF2 levels were determined using anti-TCF7L1, anti-β-catenin, and anti-RNF2 antibodies, respectively. (**b**) Rescue assay: Western blot analysis of control HeLa cells and stable RNF2 knockdown HeLa cells. TCF7L1-HA (6 μg) and myc-RNF2 plasmids (3 μg and 6 μg) were introduced into cells, which were then lysed with RIPA buffer. (**c**) HeLa cells were fixed and stained with anti-TCF7L1 (green) and anti-RNF2 (red) antibodies. Scale bar represents 10 μm. (**d**) Quantification of nuclear intensity using ImageJ after fixing the contrast value. Quantification was performed for control HeLa cells and RNF2 knockdown stable HeLa cells (n = 7 and 11). (**e**) Cycloheximide chase assay of control HeLa cells and RNF2 knockdown stable HeLa cells. The cells were treated with 10 μM cycloheximide (CHX) for 2, 4, and 8 h and lysed with RIPA buffer. (**f**) Cycloheximide chase assay of RNF2 knockdown stable HeLa cells. myc-RNF2 plasmids (6 μg) were introduced into cells, which were then treated with 10 μM cycloheximide (CHX) for 2, 4, and 8 h. Cells were lysed with RIPA buffer. (**g**) Cycloheximide chase assay of control HEK293T cells and RNF2 knockdown stable HEK293T cells. TCF7L1-HA plasmids (6 μg) were introduced into cells, which were then treated with 10 μM cycloheximide (CHX) for 2, 4, and 8 h. Cells were lysed with RIPA buffer. (**h**) In vitro ubiquitination assay was performed using RNF2 knockdown stable HeLa cells and control HeLa cells. TCF7L1-HA (6 μg) and myc-RNF2 plasmids (6 μg) were introduced into cells, followed by treatment with 10 μM MG132 for 12 h, prior to harvesting. The cells were lysed with IP buffer and subjected to immunoprecipitation with the HA antibody. Ubiquitination levels were determined by anti-UB-K48.

RNF2 knockdown stable cell lines were generated in HeLa and HEK293T cells using sgRNA-based methods, after which, the endogenous protein levels were confirmed by Western blot analysis. The amount of RNF2 protein varied in different cells, but in the RNF2 Stable knockdown cell line, the expression of RNF2 was significantly decreased (Fig. 4a). The endogenous level of TCF7L1 was dramatically increased in the RNF2 knockdown stable cell line compared to the control, indicating that TCF7L1 is continuously degraded by RNF2 through a ubiquitination-mediated process. This phenomenon was similarly observed in the TCF7L1 prominent 4T1 cell line (Fig. S2b).

Transfected RNF2 into the RNF2 knockdown stable cell line to rescue the decrease in TCF7L1 and verified that even the exogenous TCF7L1 was degraded (Fig. 4b). An immunocytochemistry assay showed that nuclear TCF7L1 was stabilized following the knockdown of RNF2 in the RNF2 knockdown stable cell line (Fig. 4c and S4a). Quantification of RNF2 expression showed that its expression was reduced in the nucleus of RNF2 knockdown stable cells, while TCF7L1 expression was dramatically increased (Fig. 4d). The relatively high level of TCF7L1 observed following MG132 treatment indicates that TCF7L1 is continuously degraded (Fig. 3b).

To investigate the effect of RNF2 knockdown on the stability of TCF7L1, a cycloheximide assay was performed on both the control (Co) and RNF2 knockdown cells. Although it was difficult to compare the results to the control due to the difference in endogenous TCF7L1 levels, TCF7L1 showed a pattern of initial decrease followed by stability (Fig. 4e). When RNF2 was transfected into the stable RNF2 knockdown cell line, the expression pattern of the control cell line was rescued and TCF7L1 destabilization was induced (Fig. 4f). Furthermore, when an equivalent amount of exogenous TCF7L1 was transfected and the cycloheximide assay was performed, the stability of TCF7L1 increased in the stable RNF2 knockdown cell line compared to the control cell line (Fig. 4g).

To confirm the change in TCF7L1 ubiquitination, a ubiquitination assay was performed. As expected, the ubiquitination level of TCF7L1 decreased significantly, providing direct evidence that RNF2 regulates the ubiquitination of TCF7L1 by acting as an E3 ligase (Fig. 4h).

### RNF2 knockdown increases TCF7L1 stability and inhibits the decrease of TCF7L1 when Wnt signaling is activated

**Figure 5:**
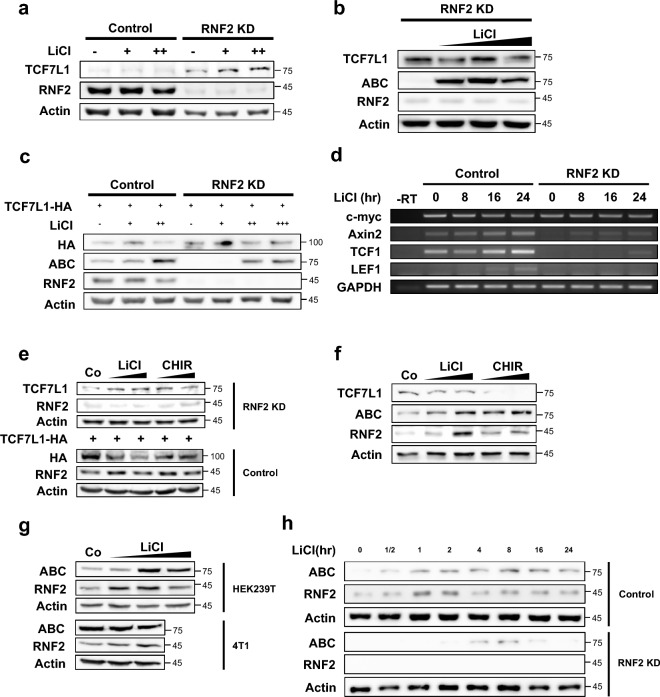
RNF2 control threshold and persistence of Wnt activation by regulating TCF7L1. (**a**) Western blot analysis of control HeLa cells and RNF2 knockdown stable HeLa cells. TCF7L1-HA (6 μg) plasmid was introduced into cells, which were then treated with LiCl (25 mM, 50 mM) conditioned media for 16 h. Cells were lysed with RIPA buffer. (**b**) Western blot analysis of RNF2 knockdown stable HeLa cells. Cells were treated with LiCl (25 mM, 50 mM, and 100 mM) conditioned media for 16 h. Cells were lysed with RIPA buffer. ABC refers to active β-catenin. (**c**) Western blot analysis of control HeLa cells and RNF2 knockdown stable HeLa cells. TCF7L1-HA (6 μg) plasmid was introduced into cells, which were then treated with LiCl (25 mM, 50 mM, and 100 mM) conditioned media for 16 h. Cells were lysed with RIPA buffer. (**d**) RT-PCR analysis of total RNAs extracted from control HeLa cells and RNF2 knockdown stable HeLa cells after treatment with LiCl (50 mM) conditioned media for 8, 16, and 24 h. Samples were subjected to cDNA synthesis and RT-PCR analysis. (**e**) Western blot analysis of control HeLa cells and RNF2 knockdown stable HeLa cells treated with LiCl (25 mM and 50 mM) and CHIR99021 (5 μM and 10 μM) conditioned media for 16 h. TCF7L1-HA (6 μg) plasmid was introduced into control HeLa cells. (**f**) Western blot analysis of control 4T1 cells treated with LiCl (25 mM and 50 mM) and CHIR99021 (5 μM and 10 μM) conditioned media for 16 h. (**g**) Western blot analysis of control HEK239T cells and 4T1 cells treated with LiCl (25 mM, 50 mM, and 100 mM) and conditioned media for 16 h. (**h**) Western blot analysis of control HeLa cells and RNF2 knockdown stable HeLa cells treated with 50 mM LiCl conditioned media for varying durations (30 min, 1 h, 2 h, 4 h, 8 h, 16 h, and 24 h).

To determine whether stable RNF2 knockdown cells can resist Wnt activation, they were treated with LiCl. Although there was a difference in the endogenous TCF7L1 levels between control and RNF2 knockdown cell line, under the conditions where the previous decrease was observed, the induction of Wnt activation did not decrease TCF7L1 expression (Fig. 5a). Even under higher Wnt activation in the stable knockdown cells, TCF7L1 did not decrease (Fig. 5b). To ensure that TCF7L1 levels remained the same, TCF7L1 was transfected using the same concentrations and LiCl was added to the control and stable RNF2 knockdown cells to induce Wnt activation. Then, TCF7L1 levels were observed and were found to decrease in the control cells due to Wnt activation; however, TCF7L1 levels did not decrease in the RNF2 knockdown stable cells (Fig. 5c). Interestingly, the expression of active β-catenin (ABC) was decreased in the stable RNF2 knockdown cells compared to the control, indicating a higher threshold for Wnt activation. Another interesting point was that even when the cells were treated with a concentration of LiCl that promoted cell death, the RNF2 knockdown stable cells were not affected and maintained normal TCF7L1 levels.

Next, RT-PCR was performed to validate whether the previous decrease in Wnt signaling activity upon RNF2 knockdown, observed in the luciferase assay (Fig. 2b), resulting in a decrease in the transcription of known Wnt target genes. The mRNA levels of known Wnt target genes: c-myc, Axin2, TCF1, and LEF1 were observed^29–35^. Indeed, a dramatic decrease in Axin2, TCF1, and LEF1 was observed in the RNF2 knockdown stable cell line (Fig. 5d), which correlated with the previous result, whereby luciferase activity was decreased (Fig. 2b). This suggests that RNF2 knockdown leads to the maintenance of high levels of TCF7L1, resulting in a decrease in the transcription of Wnt target genes.

### RNF2 modifies the Wnt response including threshold and persistence

To investigate whether Wnt activation affected RNF2, the level of RNF2 was observed after Wnt activation. Two types of Wnt activation were used: LiCl; the specific GSK3b inhibitor CHIR99021, which were cross-validated. In the condition where TCF7L1 decreased in the control cell line, TCF7L1 did not decrease in the RNF2 knockdown stable cell line (Fig. 5e). Furthermore, in the control cell line, RNF2 tended to increase upon Wnt activation.

Similarly, in the prominent TCF7L1 4T1 case, a common decrease in TCF7L1 was observed upon Wnt activation, and an increase in active β-catenin (ABC) was confirmed (Fig. 5f). Interestingly, the increase in RNF2 was clearly observed in proportion to active β-catenin, and this phenomenon was commonly observed in several cell lines (Fig. 5g). These results suggest that Wnt activation upregulates RNF2, inducing the degradation of TCF7L1. To confirm whether this increase in RNF2 was due to its regulation at the transcriptional level, RT-PCR was performed to confirm the change in RNF2 transcription upon Wnt activation. However, no change was observed at the transcriptional level (Figs. S4c, S5a,b). Interestingly, in the cell lines where TCF7L1 was not prominent, the downregulation of RNF2 dramatically repressed the transcription of the Wnt target genes, while overexpression of RNF2 did not result in any changes in the transcriptional expression of the Wnt target genes. This suggests two things: that the preexisting RNF2 sufficiently downregulated TCF7L1, and that RNF2 regulated the transcription of Wnt target gene specifically to the TCF7L1 level.

Next, to confirm the Wnt response mediated by RNF2, active β-catenin, and RNF2 levels were tracked following Wnt activation (Fig. 5h). Upon Wnt activation, an increase in active β-catenin was observed immediately. Furthermore, the previously observed increase in RNF2 was again observed. Although a decrease was noted slightly after the peak, the level of RNF2 was still higher than before Wnt was activated. In the RNF2 knockdown stable HeLa cells, the basal level of active β-catenin was lower than in the control cell line, which is hypothesized to be due to an increase in TCF7L1. Interestingly, active β-catenin increased slowly and was immediately stopped after the peak. A similar pattern was also observed in TCF7L1 prominent 4T1 cells (Fig. S2c). When the same conditions were applied to HEK293, it was confirmed that Wnt activation occurred slower and more gradually than in HeLa cells (Fig. S4b). The basal expression level of RNF2 was higher in HeLa cells than in HEK293 cells, indicating that it may affect the cellular response to Wnt activation.

### DNA-binding TCF7L1 may block the RNF2-mediated ubiquitination

The results obtained (Figs. S4c, S5a,b) were contrary to our initial hypothesis that RNF2 overexpression would decrease TCF7L1 and increase luciferase activity. Although we confirmed the degradation of free TCF7L1 by RNF2 in the nucleus, TCF7L1, which was already bound to DNA and functioning as a transcriptional repressor may be structurally inhibited from interacting with RNF2 or experiencing inhibited ubiquitination. Indeed, maintaining an appropriately low level of TCF7L1 is necessary to prevent the transcription of Wnt target genes when the Wnt signaling pathway has not been activated.

**Figure 6:**
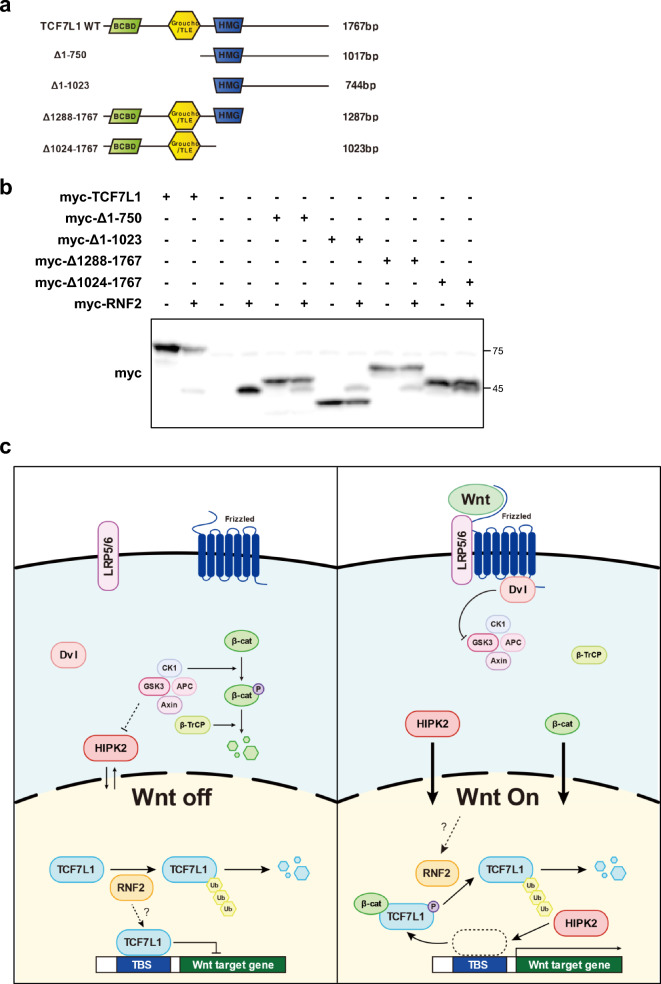
Predicted mechanism for RNF2 regulation of Wnt signaling through TCF7L1 ubiquitination. (**a**) Diagram of TCF7L1 deletion mutant used for the domain study. The image was generated using Adobe Illustrator software (version 27.3). (**b**) Western blot analysis was performed using control HeLa cells. A total of 6 μg of myc-TCF7L1 partial mutants and myc-RNF2 plasmids were introduced into cells, which were then lysed with RIPA buffer. (**c**) Proposed mechanism of the RNF2 regulation of Wnt signaling. RNF2 continuously degrades TCF7L1 through ubiquitination-mediated degradation to regulate TCF7L1 at low levels. TCF7L1 binding to a promoter region may avoid RNF2 ubiquitination. When Wnt is activated, TCF7L1 detaches from DNA and is exposed followed by the binding of ß-catenin and HIPK2 phosphorylation. Then, the TCF7L1 is degraded through RNF2-mediated ubiquitination. The image was generated using Adobe Illustrator software (version 27.3).

To confirm this, a TCF7L1 deletion mutant was constructed, centering around the DNA binding motif HMG domain, to verify if degradation occurred by RNF2 (Fig. 6a). A significant decrease was observed in the full sequence, and a decrease was also observed in the mutant where the N-terminal deletion centered around HMG, whereas no change was observed in the mutant with the C-terminal region removed (Fig. 6b). This suggests that lysine, a target for ubiquitination or important for interaction with RNF2, is located in the C-terminal region of HMG. Furthermore, this suggests that DNA-bound TCF7L1 may not be structurally influenced by RNF2.

## Discussion

Wnt signaling plays a crucial role in various biological contexts, particularly in development, where the dynamics of Wnt signaling are maximized^36–38^. Dysregulation of Wnt signaling can lead to severe phenotypes, such as abnormal morphogenesis^39^. TCF7L1 deficiency results in typical Wnt overexpression phenotypes during development, suggesting that TCF7L1 plays a critical role in the Wnt inactivation mechanism^27^. Additionally, reports suggest that TCF7L1 is involved in axis induction and mediates specific responses to Wnt/β-catenin signaling during mesoderm development^20,20^.

Until now, the known post-translational modifications to TCF7L1 upon Wnt activation are the binding to β-catenin and its phosphorylation by HIPK2, which results in its detachment from DNA^27,36,37^. In this study, we discovered a gene called RNF2 that regulates TCF7L1 levels through ubiquitination. The proposed mechanism involving this gene was investigated (Fig. 6c).

When Wnt is not activated, TCF7L1 exists in the nucleus and binds to the DNA and nuclear basal β-catenin, preventing the expression of target genes by β-catenin. Since excessive TCF7L1 in the nucleus cannot respond to Wnt cues appropriately, there is a need to maintain an appropriate level. Therefore, the remaining TCF7L1, which is not bound to nuclear DNA is continually degraded by RNF2 expressed in the nucleus, maintaining basal levels. However, when degradation is blocked by the protease inhibitor MG132, TCF7L1 levels notably increase. Upon Wnt activation, TCF7L1 is phosphorylated by β-catenin, while HIPK2 is localized to the nucleus and detaches TCF7L1 from the DNA. This process undergoes ubiquitination-mediated degradation by the E3 ligase RNF2.

Experimental domain studies of TCF7L1 deletion mutants revealed that the interaction between RNF2 and TCF7L1 is affected when TCF7L1 is bound to DNA. This suggests that detachment following Wnt activation may expose a domain that can interact with RNF2 or a ubiquitination site for TCF7L1 degradation. During Wnt activation by pt-β-catenin transfection, TCF7L1 levels did not decrease, since, unlike Wnt activation by targeting GSK3b or more upstream Wnt ligands, this activation does not affect HIPK2 activation. Although β-catenin binding has been reported to affect the interaction between TCF7L1 and HIPK2, the detachment of TCF7L1 is mainly caused by HIPK2^27^. Moreover, it was experimentally shown that binding alone was insufficient to induce a decrease in TCF7L1.

Knockdown of RNF2 hindered the continuous ubiquitination-mediated degradation of TCF7L1 in the nucleus, which led to a sharp increase in nuclear TCF7L1 levels. Furthermore, a rapid decrease in luciferase activity and transcription of Wnt target genes subsequently followed due to an increase in TCF7L1 levels. The cycloheximide assay confirmed that RNF2 reduces TCF7L1 stability, while Wnt activation itself also showed a temporary increase in RNF2 expression levels. These results establish RNF2 as a novel positive regulator of Wnt signaling.

Each cell line presents a different level of RNF2 expression, which determines the continuous degradation rate of TCF7L1 and the basal TCF7L1 levels. The effects of RNF2 expression levels were also observed in the TCF7L1 ubiquitination assay, where RNF2 overexpression led to an increase in ubiquitinated TCF7L1. Additionally, this mechanism is conserved in both prominent and non-prominent TCF7L1 cell lines. In the case of 4T1, which presents a significant expression of RNF2, the prominent level of TCF7L1 suggests that Wnt activation itself may increase RNF2 or activate it through an unknown mechanism.

The candidates confirmed by mass spectrometry represented genes that are highly likely to interact with TCF7L1, and therefore, have the potential to play an essential role in the development process by influencing TCF7L1. RT-PCR confirmed that most of these genes are expressed during development (Fig. S6a). RNF2 exhibited a similar mRNA expression pattern to TCF7L1 during embryo development, and the organs in which they were expressed were also similar (Fig. S6c,d). Furthermore, in situ hybridization confirmed spatial and temporal expression overlap between TCF7L1 and RNF2 (Fig. S6e), suggesting that the regulatory mechanism of TCF7L1 by RNF2 may be conserved during embryo development. When RNF2 mRNA was overexpressed through microinjection, it induced embryonic death at the gastrula stage and resulted in axis malformation as the primary phenotype, which typically occurs when there are problems with mesoderm differentiation during the process of gastrulation (Fig. S6f). However, further studies are needed to fully understand this mechanism.

Mass spectrometry analysis revealed that RING1 and RNF2 (RING1b) are among the top-ranked E3 ligases in the E3 ligase list. Both E3 ligases are well-known components of the Polycomb repressive complex 1 (PRC1) and are involved in the transcriptional repression of various genes, including development and cell proliferation, through histone H2A monoubiquitination^40–44^. Therefore, an initial hypothesis was that the level of TCF7L1 could be regulated through histone modification or changes in Wnt target gene expression.

There are reports related to the association between PRC1 and Wnt signaling^45–47^. However, in the observed ubiquitination of TCF7L1, RNF2 directly increased protein ubiquitination, which induced degradation through K48 ubiquitination, unlike epigenetic changes through histone modification. Moreover, if we expect epigenetic changes, overexpression of RNF2 should result in changes in the transcription of Wnt target genes. However, no changes were observed. Recently, RNF2 has been reported to be involved in polyubiquitination^48,49^. This appears to be an independent event, requiring further investigation in future studies.

TCF7L1 is a transcription factor that plays a critical role in various cellular processes such as stem cell differentiation and carcinogenesis^50,51^. To better understand the mechanism involved in the regulation of TCF7L1 in these processes, it is important to investigate the context in which TCF7L1 functions. Specifically, it is necessary to examine the regulatory role of RNF2 in TCF7L1-mediated breast cancer metastasis, as well as to explore whether RNF2-mediated regulation of TCF7L1 occurs only at the cellular level or requires other components.

In addition, at the cellular level, it has been difficult to observe patterns in the reduction of RNF2, except for the use of artificially generated RNF2 knockdown cell lines. Further research is required to understand RNF2-related mechanisms when TCF7L1 expression is increased to the point of Wnt signaling termination. It is anticipated that there may be specific mechanisms whereby RNF2 is degraded by specific proteins or its interaction with TCF7L1 is inhibited. Therefore, further research is needed to explore these mechanisms and their potential significance in TCF7L1 regulation.

Our results suggest that RNF2 regulates Wnt signaling by controlling the expression of TCF7L1, indicating a dependency of TCF7L1 expression on RNF2. These findings provide insight into the role of RNF2 in Wnt signaling and provide a basis for future investigations into the mechanisms underlying the regulation of Wnt signaling by RNF2.

## Materials and methods

### Plasmids

To generate the pcDNA3.1 TCF7L1 BirA-HA vector for use in BioID mass spectrometry experiments, the TCF7L1 insert was amplified from the pCMV-SPORT6-TCF7L1 plasmid (RIKEN clone ID : IRAK119L17) using PCR with primers designed to include appropriate restriction enzyme sites for cloning into the pcDNA3.1 MCS-BirA(R118G)-HA vector (Addgene #36047).

RNF2 was amplified from HeLa cDNA and inserted into pCS2 + 6myc vector.

To generate TCF7L1 mutants, TCF7L1 was amplified from HeLa cDNA and inserted into pCS2 + 6myc vector. The resulting plasmids were named as follows: pCS2 + 6myc-TCF7L1, pCS2 + 6myc-TCF7L1Δ1-750, pCS2 + 6myc-TCF7L1Δ1-1023, pCS2 + 6myc-TCF7L1Δ1288-1767, and pCS2 + 6myc-TCF7L1Δ1024-1767.

### Cell culture and transfection

HeLa, HEK293T and 4T1 cells were obtained from ATCC.

HeLa and HEK293T cell lines were cultured in Dulbecco's Modified Eagle's Medium (DMEM, Corning) supplemented with 10% fetal bovine serum (FBS, Corning), 100 U/mL penicillin, and 100 μg/mL streptomycin in a humidified incubator at 37 °C and 5% CO_2_. Cells were passaged using trypsinization when they reached 80–90% confluence.

The 4T1 cell line was cultured in RPMI-1640 medium (Hyclone) supplemented with 10% fetal bovine serum (FBS, Corning), 100 U/mL penicillin, and 100 μg/mL streptomycin in a humidified incubator at 37 °C and 5% CO_2_. Cells were passaged using trypsinization when they reached 80–90% confluence.

The cells were maintained and transfected without any contamination.

For transient transfection, cells were seeded in 100 mm plates and grown to 70–80% confluency. Transfections were performed using Lipofectamine 2000 (Invitrogen) according to the manufacturer’s instructions. Briefly, DNA (plasmid or siRNA) and transfection reagent were separately diluted in serum-free medium, mixed, and added to the cells. To ensure equal amounts of transfection reagent, an empty vector was added during the transfection process. After incubation for the specified period, the transfection mixture was replaced with complete growth medium.

For stable knockdown cell generation, cells were seeded in 6-well plates and grown to 50–60% confluency. Transfection was performed using Lipofectamine 2000 (Invitrogen) according to the manufacturer’s instructions.

### In-gel digestion and LC−MS/MS analysis

The process involved culturing HeLa cells in an appropriate medium until they reach 70–80% confluency, transfecting the cells with TCF7L1-BirA plasmid, and adding biotin to the cell culture media to a final concentration of 50 µM to enable biotinylation of proximal proteins. After 16 h, the cells were treated with 10 µM of the proteasome inhibitor MG132 for 6 h to prevent the degradation of biotinylated proteins. Streptavidin beads were added to the cell lysate, and the mixture was incubated at 4 °C with gentle shaking for 4 h to allow biotinylated proteins to bind to the beads. Eluted proteins from streptavidin were analyzed by further mass spectrometry process.

Gel fraction and enzyme digestion were performed as described previously^52^. Gels were divided and sliced into seven fractions according to molecular weight. Sliced gels were washed and destained with a 30% methanol and destaining solution (10 mM ammonium bicarbonate and 50% acetonitrile). After drying, gels underwent reduction with 10 mM dithiothreitol and alkylation of cysteines with 55 mM iodoacetamide. After the gels were washed with distilled water, tryptic digestion was performed in 50 mM ammonium bicarbonate at 37 °C for 12–16 h. Tryptic peptides were obtained by two extraction steps in 50 mM ammonium bicarbonate and 50% acetonitrile containing 5% trifluoroacetic acid. The resulting peptide extracts were pooled and lyophilized in a vacuum concentrator and stored at 4 °C.

Liquid chromatography with tandem mass spectrometry (LC–MS/MS) analysis was performed according to the methodology presented in a previous report^53^. Tryptic digestide samples were dissolved with 0.5% trifluoroacetic acid prior to further analysis. 5 μL dissolved samples were transferred into a 100 μm × 2 cm nanoViper trap column and a 15 cm × 75 μm nanoViper analysis column (Thermo Fisher Scientific) at a flow rate of 300 nL/min and were eluted with a gradient of 5%–40% acetonitrile over 95 min. All MS and MS/MS spectra captured by the Q Exactive Plus mass spectrometer (Thermo Fisher Scientific) were acquired in data-dependent top 12 mode. Peptide identification was based on monoisotopic mass selection, precursor mass tolerance of ± 5 Da, fragment mass tolerance of ± 0.8 Da, two missed cleavage and fixed modification of carbamidomethyl cysteine. Individual spectra were accepted based on a Mascot ion threshold score of 0.05 that is calculated specifically for each database search. Peptide validator was used to calculate the false discovery rates (FDR) at 1.0% for peptide and protein matches above the identity threshold.

### Dual Luciferase reporter assay

Cells were transfected with a luciferase reporter plasmid (200 ng) containing TOPFlash promoter, along with a control Renilla luciferase plasmid (50 ng) to normalize for transfection efficiency. Transfections were performed using Lipofectamine 2000 (Invitrogen) according to the manufacturer's instructions.

Cells were harvested 24–48 h post-transfection and luciferase activity was measured using a dual-luciferase reporter assay system (Promega) on a GloMax luminometer (Promega). Firefly luciferase activity was normalized to Renilla luciferase activity to account for differences in transfection efficiency. For each experiment, at least three independent transfections were performed in triplicate.

### RNA interference and CRISPR/Cas9-mediated gene silencing

HEK293T cells were transfected with packaging plasmids delta 8.9 and VSV-G, and the shRNA expression plasmid (pLKO.1-TRC) targeting the gene of interest using Lipofectamine 2000 (Invitrogen). After 48 h, the lentiviral particles contained media were harvested from the culture medium by filtering through a 0.45 µm filter.

Target cells were seeded onto 6-well plates and infected with lentiviral particles. After 24 h, the culture medium was replaced with fresh medium containing 1 µg/mL puromycin (Sigma-Aldrich) to select for cells that have integrated the shRNA expression plasmid. the shRNA sequence was designed to target the gene of interest using available online tools such as GPP Web Portal (https://portals.broadinstitute.org/gpp/public/).

Primers used for shRNA were described in Table 3.

The validation of knockdown efficiency was performed in cells that survived puromycin selection. To confirm the knockdown efficiency, mRNA levels of the gene of interest were analyzed by quantitative real-time PCR (qRT-PCR). Total RNA was isolated using TRIzol reagent (Thermo Fisher Scientific) and reverse-transcribed into cDNA using a reverse transcription kit (Thermo Fisher Scientific). qRT-PCR was performed using SYBR Green PCR Master Mix (Thermo Fisher Scientific) and gene-specific primers on a real-time PCR machine (Bio-Rad).

Primers used for knockdown validation were described in Table 4.

The PX459 plasmid vector (Addgene #62988) was used to express the guide RNA (gRNA) for genome editing using the CRISPR/Cas9 system. To generate stable knockout or knockdown cell lines, the gRNA sequence was designed to target the gene of interest using available online tools such as CHOPCHOP (https://chopchop.cbu.uib.no/). The gRNA sequence was then cloned into the PX459 vector using standard molecular biology techniques. The vector was linearized using the BbsI restriction enzyme, and the gRNA sequence was ligated into the vector using T4 DNA ligase.

The PX459 vector expressing the gRNA sequence was transfected into the target cells using Lipofectamine 2000 (Invitrogen) according to the manufacturer's instructions. Cells were selected for stable integration of the gRNA expression cassette by culturing in medium containing puromycin (1 µg/mL) for 3–5 days.

The efficiency of the genome editing was confirmed by analyzing the mRNA pattern and protein levels of the target gene by Mutation detection and Western blot, respectively. Total RNA was extracted using TRIzol reagent (Thermo Fisher Scientific) and reverse-transcribed into cDNA using a reverse transcription kit (Thermo Fisher Scientific). Mutation detection was performed using EnGen^®^ Mutation Detection Kit (NEB) according to the manufacturer's instructions. The image was captured using GelDoc-It imaging system (UVP).

Primers used for making gRNA included PX459 were described in Table 5. Primers used for mutation detection were described in Table 6.

Protein lysates were prepared using RIPA buffer (50 mM Tris–HCl, pH 7.5, 150 mM NaCl, 1% NP-40, 0.5% sodium deoxycholate, and 0.1% SDS) supplemented with protease inhibitors. Protein concentration was determined using the Bradford reagent. Western blot was performed using appropriate primary and secondary antibodies.

### Western blot

Cells were harvested and lysed in RIPA buffer (20 mM Tris–Cl, pH 7.5, 1% Triton X-100, 0.1% SDS, 150 mM NaCl, 1 mM EDTA, 1 mM EGTA, 1 mM β-glycerophosphate, 1 mM Na3VO4) supplemented with cOmplete Protease Inhibitor Cocktail (Roche). Total protein concentration was determined using the Bradford reagent. Equal amounts of protein were loaded onto a 10% SDS-PAGE gel and separated by electrophoresis. The separated proteins were transferred to a PVDF membrane (Merck) using a wet transfer system (Bio-Rad).The membrane was then blocked with 5% skim milk in TBST (Tris-buffered saline with 0.1% Tween 20) for 1 h at room temperature.

Before antibody hybridization, the membrane was cut according to the size of the protein ladder (PM2610) to ensure precise size determination. All original data in the supplemental information cover the sizes of the cut membranes. After blocking, the membrane was incubated with the primary antibody against the target protein (dilution in 5% BSA/TBST) overnight at 4 °C. The following day, the membrane was washed three times with TBST and incubated with the secondary antibody conjugated with horseradish peroxidase (HRP) (1:1000 dilution in 5% skim milk/TBST) for 1 h at room temperature.

In cases where signal intensity was low, SuperSignal West Pico PLUS (Thermo Scientific) was employed for protein detection instead of enhanced chemiluminescence (ECL) reagents (Bio-Rad). The membrane was washed with TBST, and the protein of interest was visualized using the SuperSignal substrate according to the manufacturer's instructions. Subsequently, the protein bands were captured and analyzed using a LAS-4000 imaging system.

Antibody information used for western blot was described in Table 7.

### Immunoprecipitation

Cells were harvested and lysed in IP buffer (50 mM Tris–HCl, pH 7.5, 150 mM NaCl, 1% NP-40, 1 mM EDTA, and protease inhibitors). Protein concentration was determined using the Bradford reagent.

Equal amounts of protein were incubated with the primary antibody against the protein of interest (1:100 dilution in IP buffer) overnight at 4 °C with gentle shaking. The following day, Protein G magnetic beads (Thermo Fisher Scientific) were added to the lysate and incubated for 1 h at 4 °C with gentle shaking. The beads were then washed three times with IP buffer and eluted with SDS sample buffer (62.5 mM Tris–HCl, pH 6.8, 10% glycerol, 2% SDS, 0.01% bromophenol blue, and 5% β-mercaptoethanol). The eluted proteins were separated by SDS-PAGE and analyzed by Western blot using the appropriate primary and secondary antibodies.

### Immunofluorescence

Cells were seeded onto coverslips and fixed with 4% paraformaldehyde in phosphate-buffered saline (PBS) for 10 min at room temperature. The fixed cells were permeabilized with 0.1% Triton X-100 in PBS for 10 min and blocked with 5% bovine serum albumin (BSA) in PBS for 1 h at room temperature.

Primary antibodies against the protein of interest and the cellular component of interest were added simultaneously to the coverslips (1:100 dilution in 1% BSA/PBS) and incubated overnight at 4 °C. The following day, coverslips were washed three times with PBS and incubated with fluorescently-labeled secondary antibodies against the primary antibodies (Alexa 488 and 594, Invitrogen, 1:400 dilution in 1% BSA/PBS) for 1 h at room temperature. Hoechst was added to the coverslips to stain the nucleus.

After three washes with PBS, coverslips were mounted onto glass slides using fluorescent mounting medium. Images were acquired using a fluorescence microscope using FV31S software (Olympus). Quantification was performed using ImageJ software.

### Total RNA isolation and RT-PCR

The cells were harvested using trypsin–EDTA and pelleted by centrifugation, and the supernatant was removed. The cells were washed with sterile 1X phosphate-buffered saline (PBS) and pelleted again. The PBS was removed, and the cells were lysed with Trizol reagent. The lysate was incubated at room temperature, and chloroform was added to the mixture. After incubating the mixture, it was centrifuged, and the aqueous phase was transferred to a new tube. Isopropanol was added to the tube to precipitate the RNA, and the tube was centrifuged again. RNA pellet was washed with 75% ethanol, air-dried, and dissolved in RNase-free water. RNA concentration and purity were measured using a NanoDrop spectrophotometer. Primers used for RT-PCR were described in Table 8.

To isolate total RNA from *Xenopus* embryos at developmental stage 11, Trizol reagent was used to lyse the embryos, followed by centrifugation with chloroform to separate the aqueous phase containing the RNA, and isopropanol was added to the aqueous phase to precipitate the RNA, which was then washed with ethanol and dissolved in RNase-free water for downstream applications.

Primers used for RT-PCR were described in Table 9.

### *Xenopus* embryos and injection

The female and male *Xenopus laevis* were obtained from Nasco and Hallym university, and used in accordance with the guidelines provided by the POSTECH IACUC (Institutional Animal Care and Use Committees) in Korea, who certified that the animals were being handled ethically.

The *Xenopus laevis* eggs were collected and fertilized as previously described in a previous report, and microinjections were performed following the protocol outlined in the same report^54^. The developmental stages of the embryos were determined based on the system described by Zahn et al.^55^. The injections were carried out at the 4-cell stage of the embryo, targeting the dorsal animal part.

The complete coding sequence of *Xenopus laevis* RNF2.L was amplified from tadpole stage embryos through PCR and subsequently cloned into the pCST + vector. Capped mRNA was synthesized from NotI-linearized plasmids using the mMESSAGE mMACHINE SP6 Transcription Kit (Invitrogen).

### In situ hybridization

Whole-mount in situ hybridization (WISH) was carried out following the protocol described by Harland^56^.

Primers used for cloning probes were described in Table 10.

### Statistical analysis

Each experiment was repeated at least three times to ensure reproducibility. The error bars displayed indicate the standard deviation (SD) of the data obtained from at least three independent experiments. The statistical significance was determined using a two-tailed student's t-test, and the levels of significance are indicated by asterisks: (n.s. P > 0.05, *P ≤ 0.05, **P ≤ 0.01, ***P ≤ 0.001).

### Ethical approval

All methods and procedures were carried out in accordance with relevant guidelines and regulations. This study was approved by the Institutional Animal Care and Use Committee (IACUC) of Pohang University of Science and Technology with the approval number POSTECH-2022–0139. Our study was conducted in accordance with the ARRIVE guidelines (https://arriveguidelines.org), which provide a set of recommendations for reporting experiments involving animal subjects. This ensures transparency and compliance with ethical standards in reporting our experimental results.

### Supplementary Information

Supplementary Figures.

Supplementary Tables.

## Data Availability

All data generated or analyzed during this study are included in this published article and its supplementary information files.
