# Supplementary material for: RNF2 regulates Wnt/ß-catenin signaling via TCF7L1 destabilization
Source: Sci Rep. 2023 Nov 13;13:19750. doi: 10.1038/s41598-023-47111-x (PMC10643375; doi:10.1038/s41598-023-47111-x)
Supplement: Supplementary file 1 — Supplementary Figures. [file 41598_2023_47111_MOESM1_ESM.pdf]

Supplementary Information

15 Supplementary figures with legends

RNF2 regulates Wnt/ß-catenin signaling via TCF7L1 destabilization

Youngmu Koo[1], Wonhee Han[2], Byeong-Rak Keum[1], Leila Lutz[1], Sung Ho Yun[3], Gun-Hwa Kim[4], Jin-Kwan Han[1,*]

1. Department of Life Sciences, Pohang University of Science and Technology, 77 Cheongam-Ro, Nam-Gu, Pohang, Gyeongbuk, 37673, Korea.

2. F. M. Kirby Neurobiology Center, Boston Children's Hospital, Department of Neurology, Harvard Medical School, Boston, MA 02115, USA.

3. Center for Research Equipment, Korea Basic Science Institute, Cheongju 28119, Republic of Korea

4. Research Center for Bioconvergence Analysis, Korea Basic Science Institute, Cheongju 28119, Republic of Korea

* Corresponding author: jkh@postech.ac.kr

Figure S1

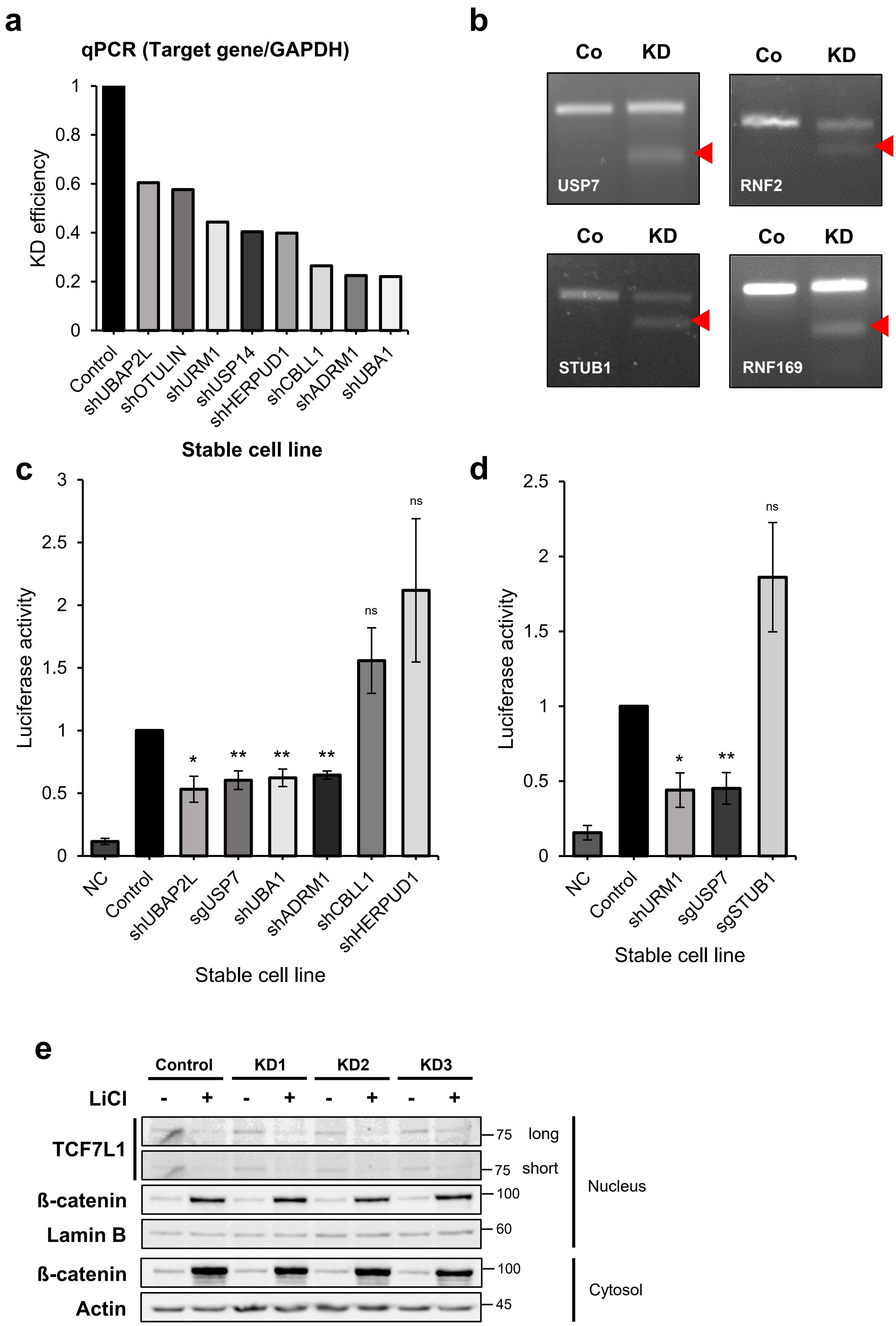
a
qPCR (Target gene/GAPDH)
KD efficiency
1
0.8
0.6
0.4
0.2
0
Control
shUBAP2L
shOTULIN
shURM1
shUSP14
shHERPUD1
shCBLL1
shADRM1
shUBA1
Stable cell line
b
Co
KD
USP7
RNF2
STUB1
RNF169
c
Luciferase activity
3
2.5
2
1.5
1
0.5
0
NC
Control
shUBAP2L
sgUSP7
shUBA1
shADRM1
shCBLL1
shHERPUD1
*
**
ns
Stable cell line
d
Luciferase activity
2.5
2
1.5
1
0.5
0
NC
Control
shURM1
sgUSP7
sgSTUB1
*
**
ns
Stable cell line
e
Control
KD1
KD2
KD3
LiCl
-
+
TCF7L1
ß-catenin
Lamin B
Actin
75
long
short
100
60
45
Nucleus
Cytosol

Figure S2

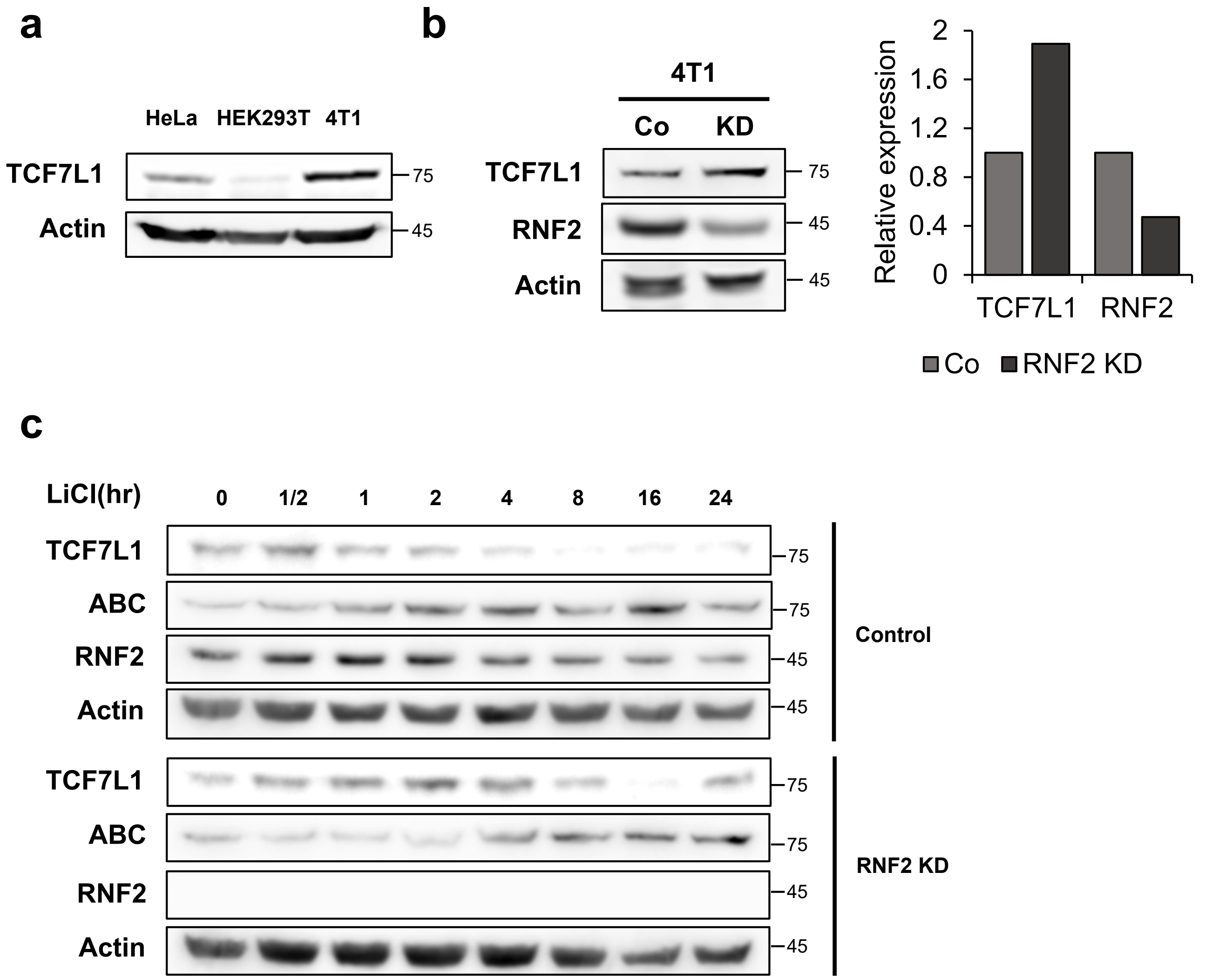
a
HeLa HEK293T 4T1
TCF7L1
Actin
75
45
b
4T1
Co KD
TCF7L1
RNF2
Actin
75
45
45
Relative expression
2
1.6
1.2
0.8
0.4
0
TCF7L1 RNF2
Co RNF2 KD
c
LiCl(hr)
0 1/2 1 2 4 8 16 24
TCF7L1
ABC
RNF2
Actin
Control
RNF2 KD

Figure S3

a

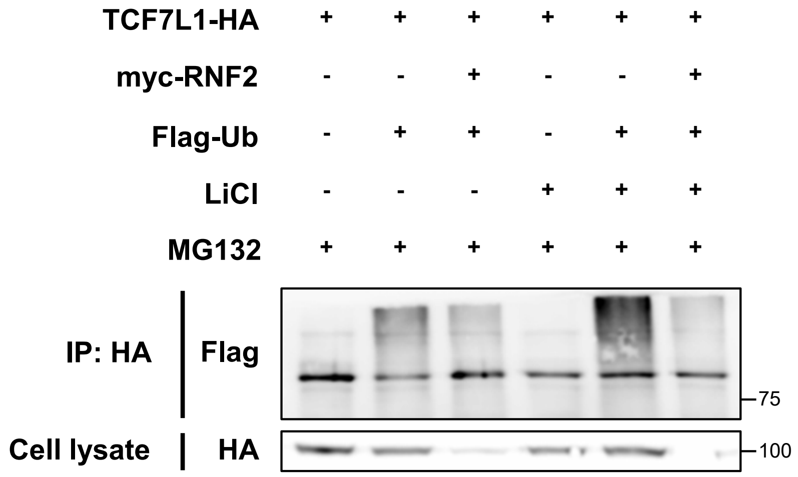
TCF7L1-HA + + + + + +
myc-RNF2 - - + - - +
Flag-Ub - + + - + +
LiCl - - - + + +
MG132 + + + + + +
IP: HA
Flag
75
Cell lysate
HA
100

Figure S4

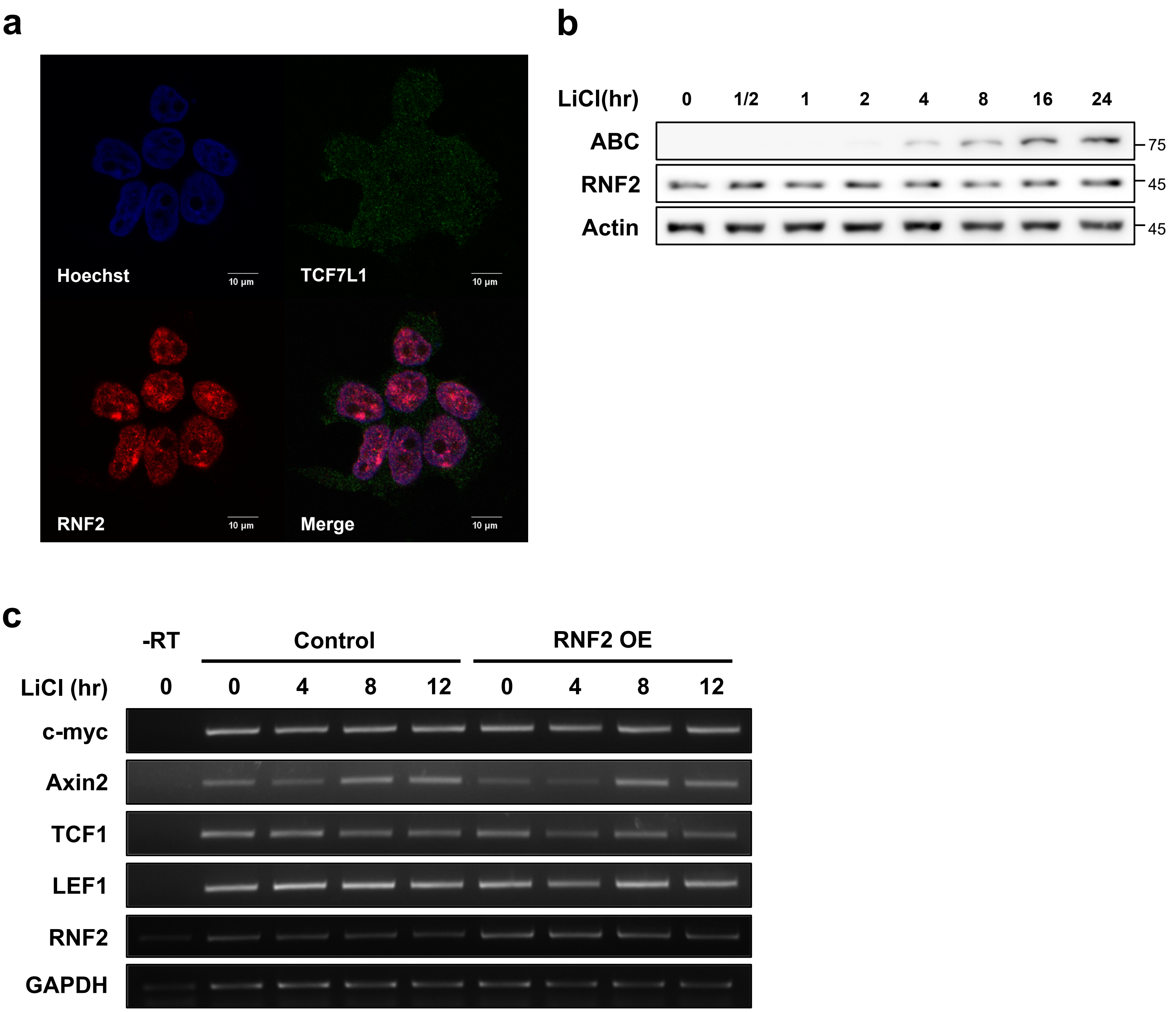

a
Hoechst
TCF7L1
RNF2
Merge
10 µm
b
LiCl(hr)
0
1/2
1
2
4
8
16
24
ABC
RNF2
Actin
75
45
45
c
-RT
Control
RNF2 OE
LiCl (hr)
0
0
4
8
12
0
4
8
12
c-myc
Axin2
TCF1
LEF1
RNF2
GAPDH

Figure S5

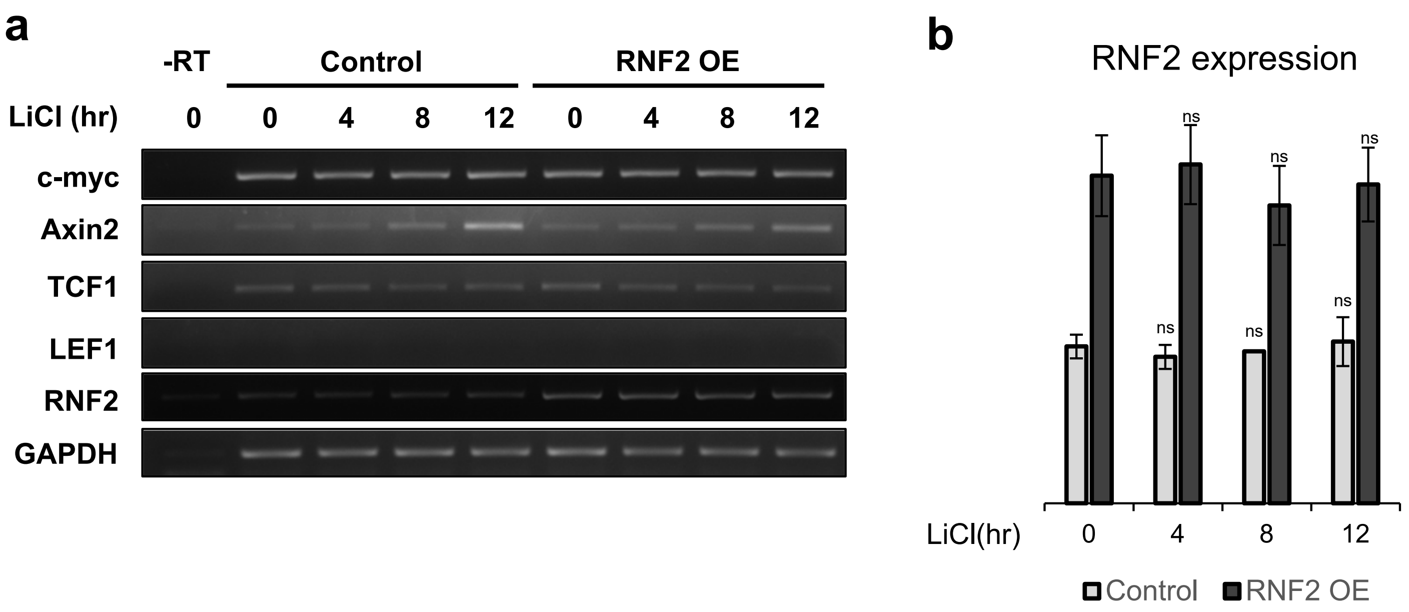
a
-RT
Control
RNF2 OE
LiCl (hr)
0
0
4
8
12
0
4
8
12
c-myc
Axin2
TCF1
LEF1
RNF2
GAPDH
b
RNF2 expression
ns
ns
ns
ns
ns
ns
LiCl(hr)
0
4
8
12
Control
RNF2 OE

Figure S6

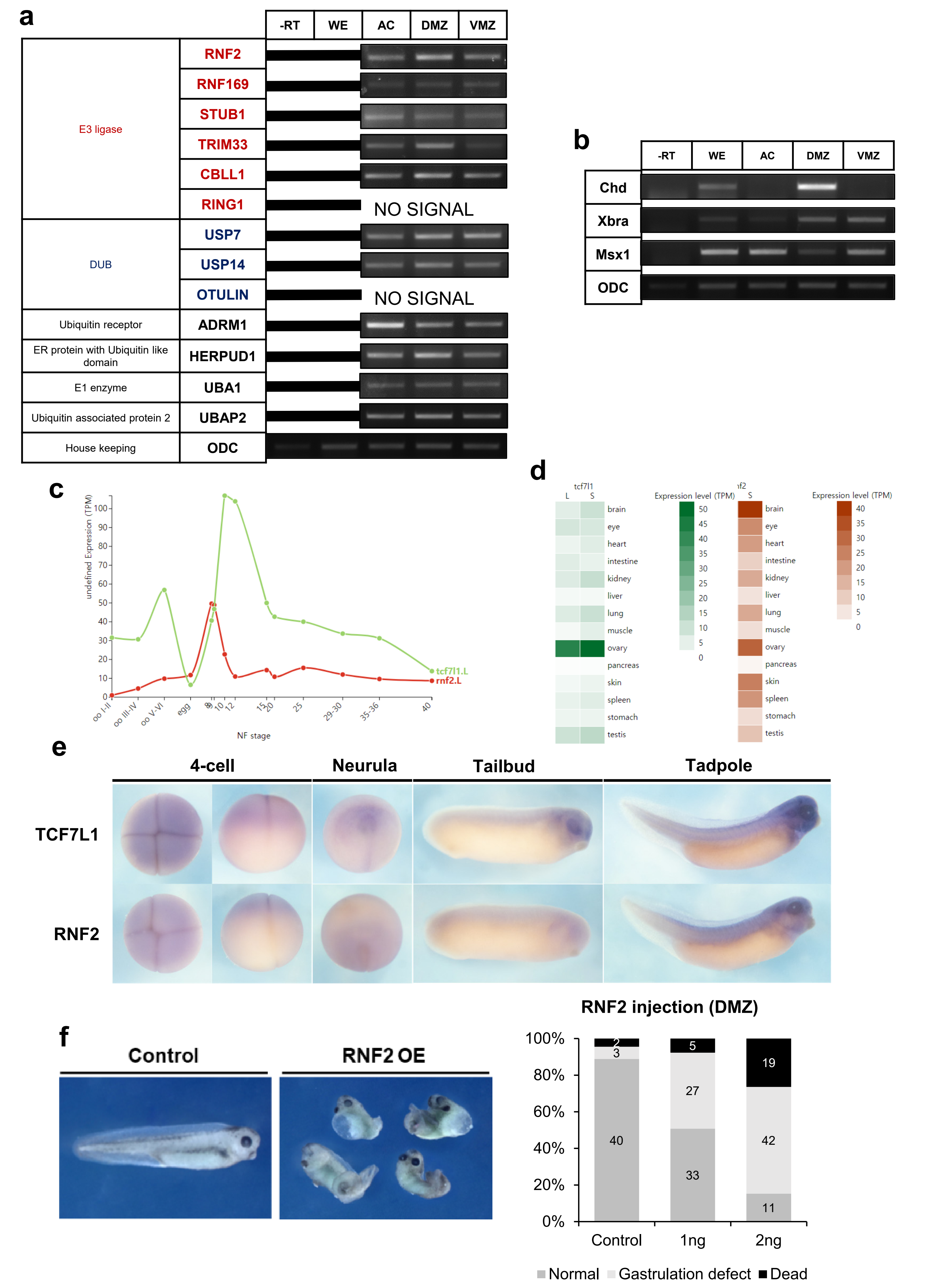

a
-RT
WE
AC
DMZ
VMZ
E3 ligase
RNF2
RNF169
STUB1
TRIM33
CBLL1
RING1
NO SIGNAL
DUB
USP7
USP14
OTULIN
NO SIGNAL
Ubiquitin receptor
ADRM1
ER protein with Ubiquitin like domain
HERPUD1
E1 enzyme
UBA1
Ubiquitin associated protein 2
UBAP2
House keeping
ODC
b
Chd
Xbra
Msx1
ODC
c
undefined Expression (TPM)
NF stage
tcf7l1.L
rnf2.L
d
tcf7l1
L
S
Expression level (TPM)
brain
eye
heart
intestine
kidney
liver
lung
muscle
ovary
pancreas
skin
spleen
stomach
testis
e
4-cell
Neurula
Tailbud
Tadpole
TCF7L1
RNF2
f
Control
RNF2 OE
RNF2 injection (DMZ)
100%
80%
60%
40%
20%
0%
Control
1ng
2ng
Normal
Gastrulation defect
Dead

Figure S7

Figure 1a

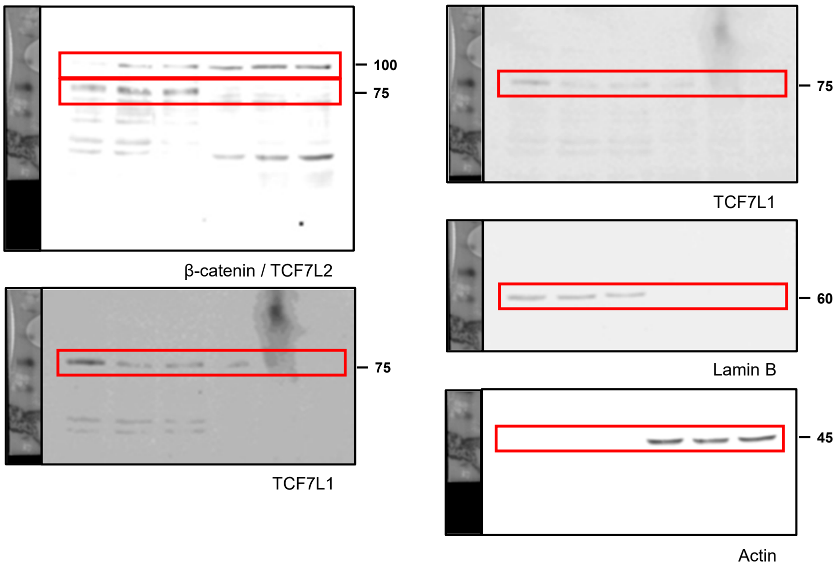

Figure 1b

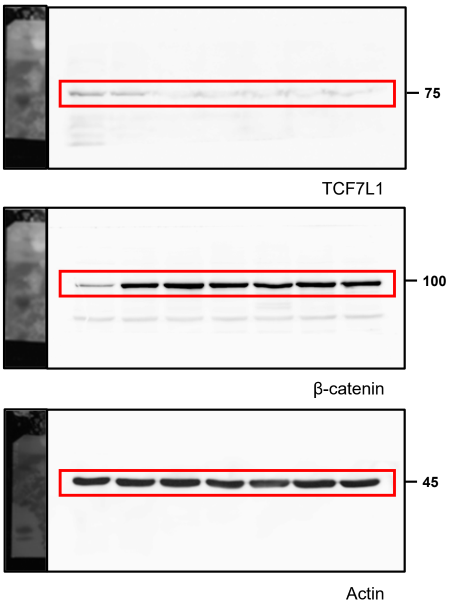

Figure 1c

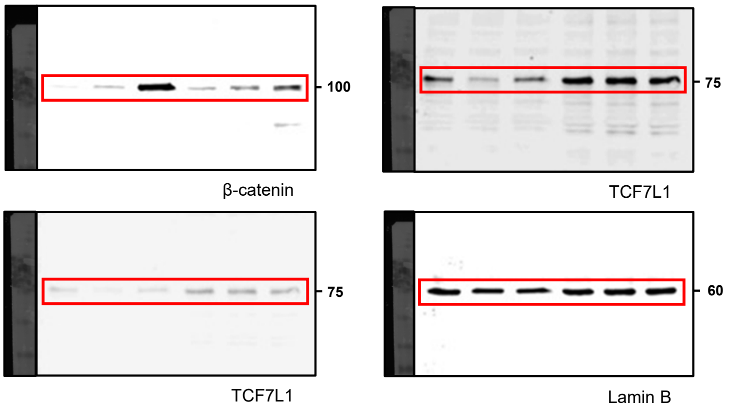

Figure 1d

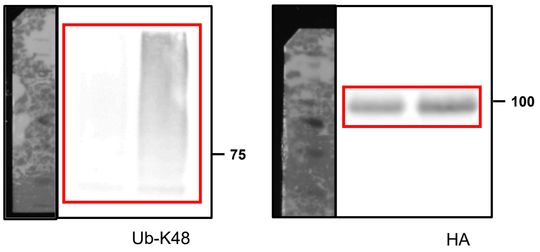

Figure 3a

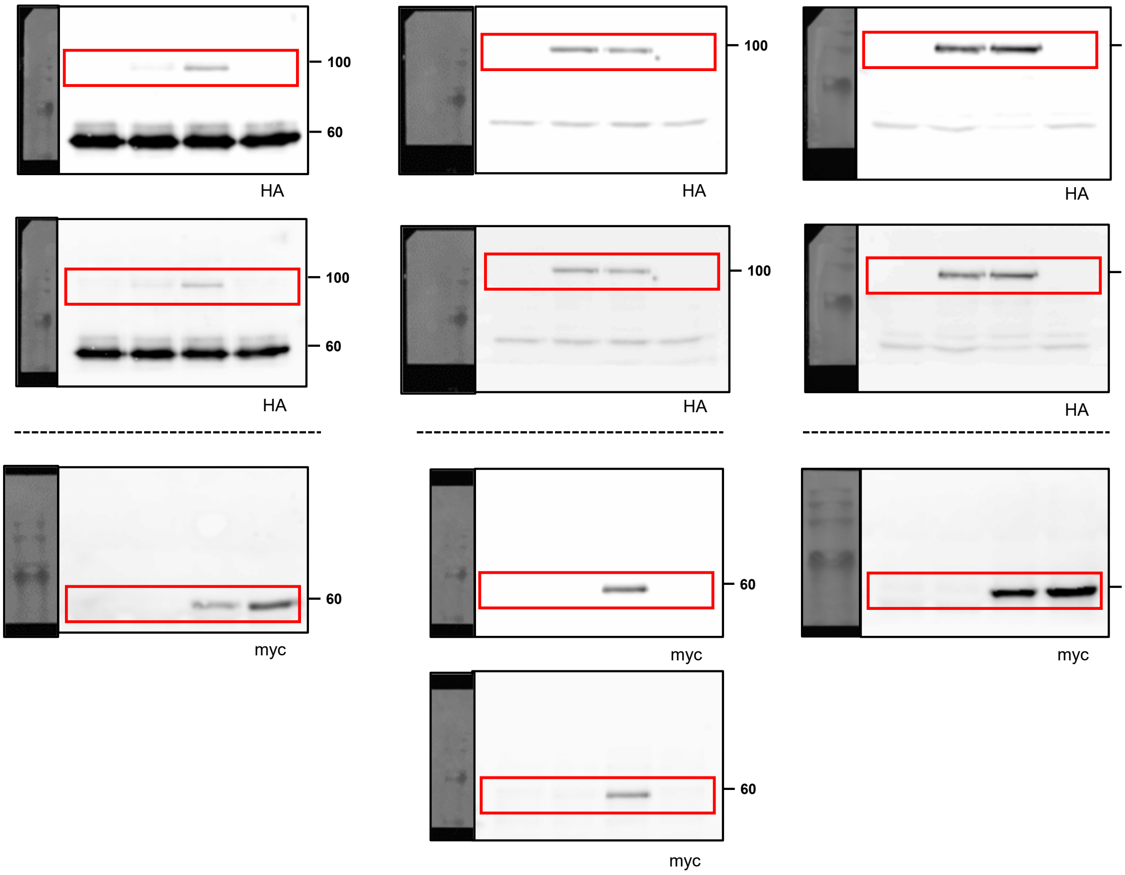

Figure 3c

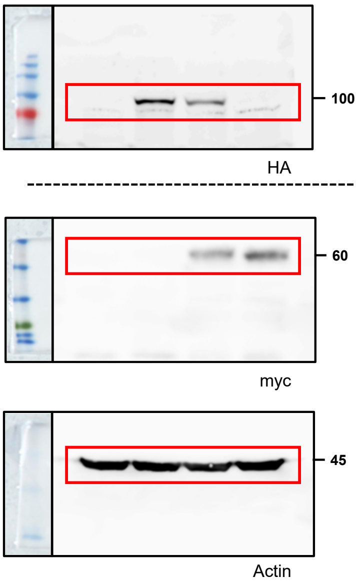

Figure S8

Figure 3d

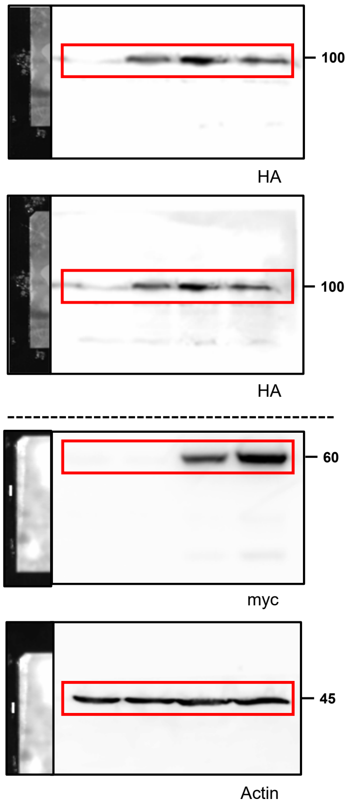

Figure 3e

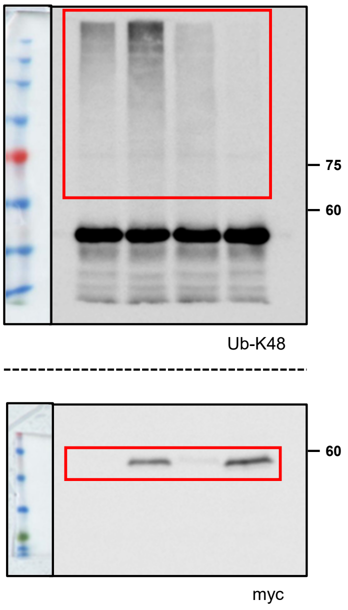

Figure 3f

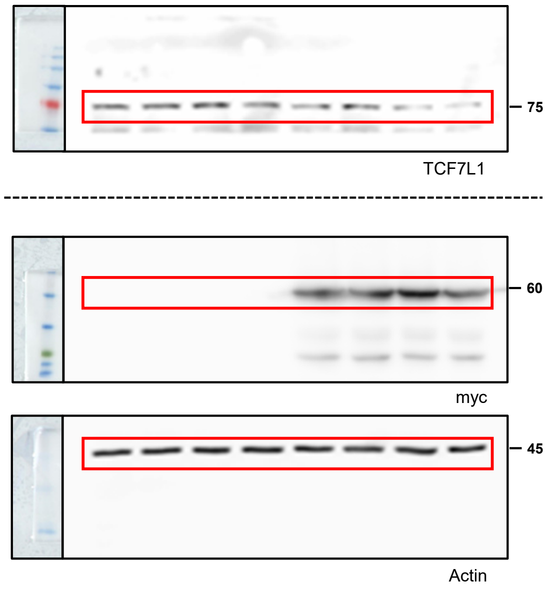

Figure 4b

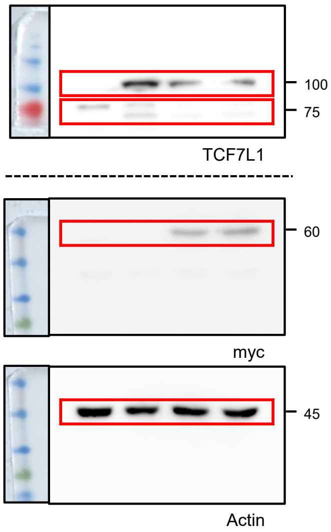

Figure 4e

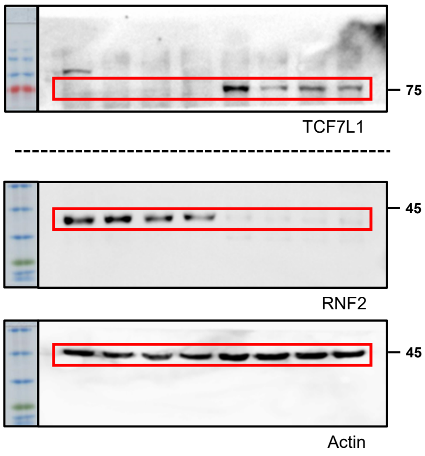

Figure 4a

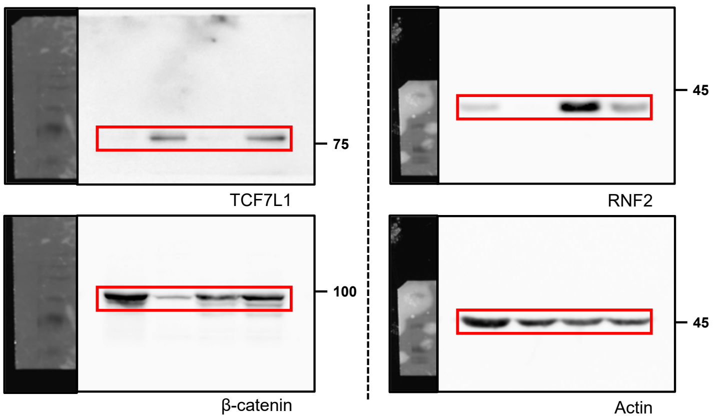

Figure 4f

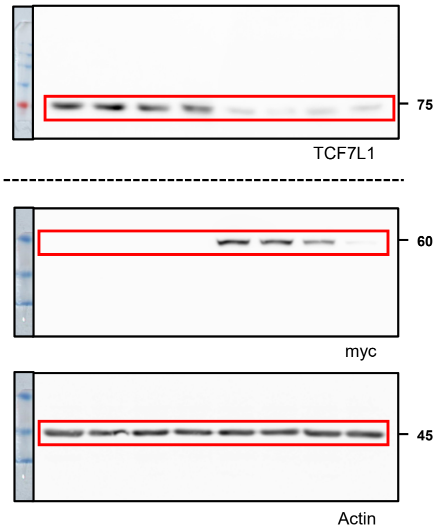

Figure 4g

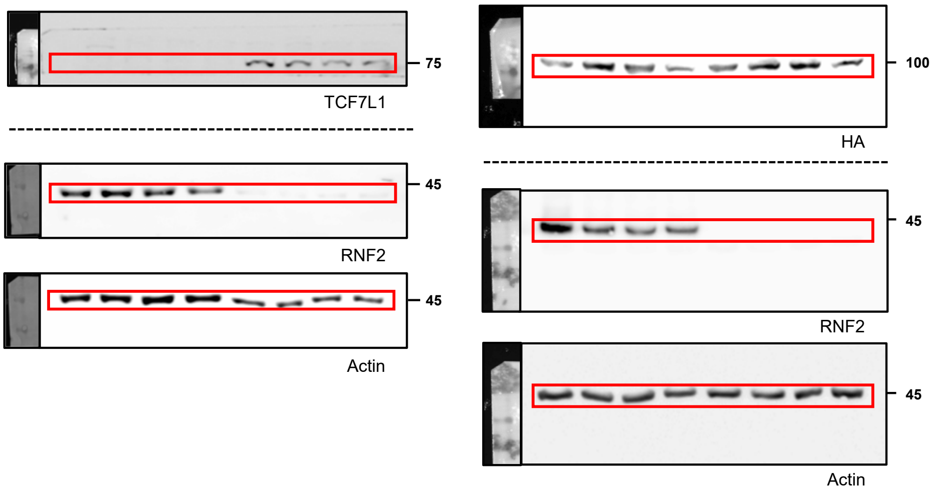

Figure S9

Figure 4h

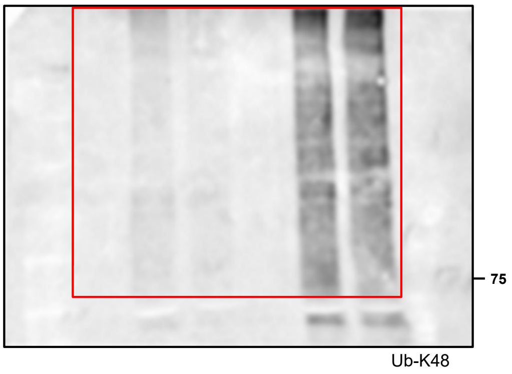

Figure 5a

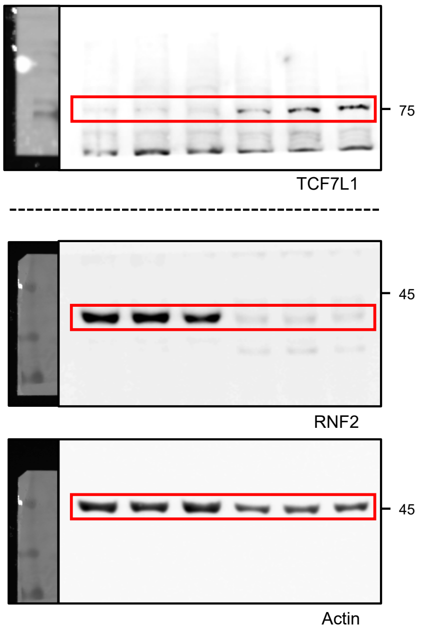

Figure 5b

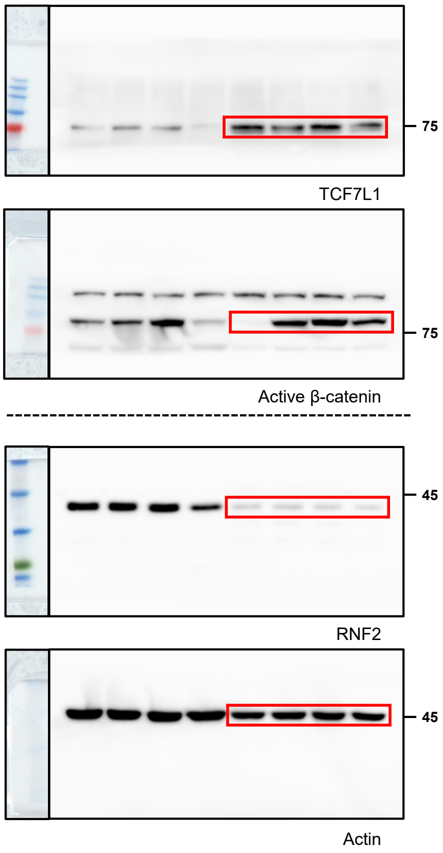

Figure 5c

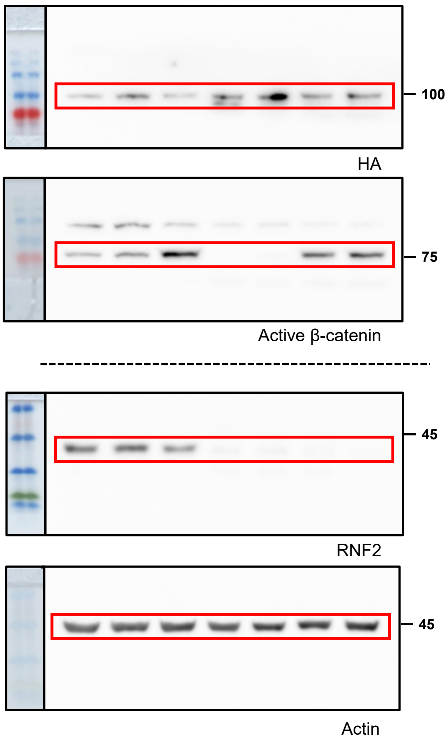

Figure 5e

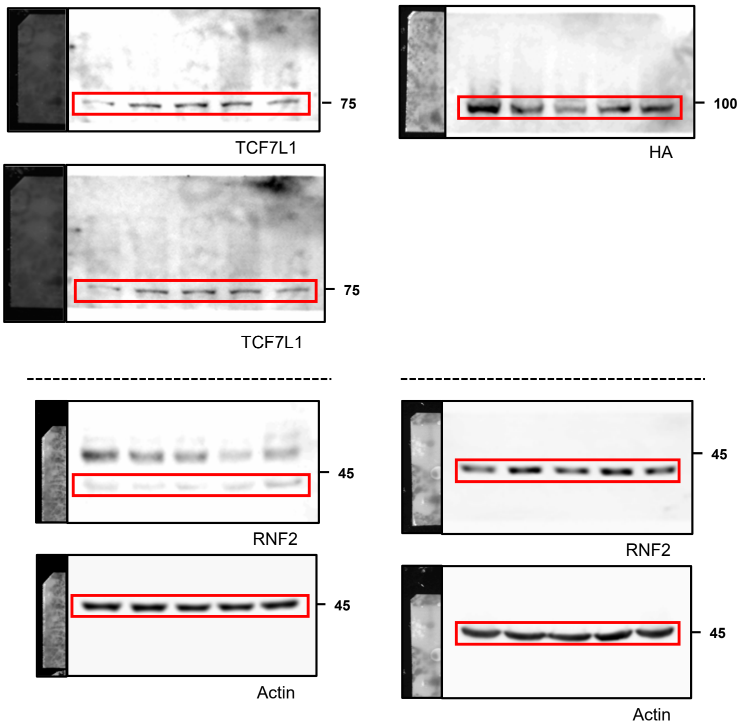

Figure 5f

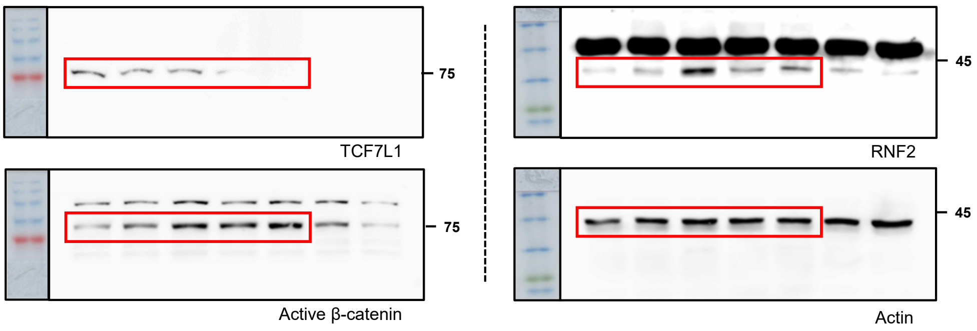

Figure S10

Figure 5g

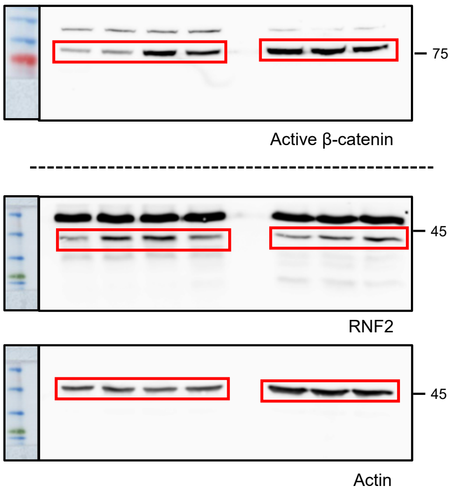

Figure 5h

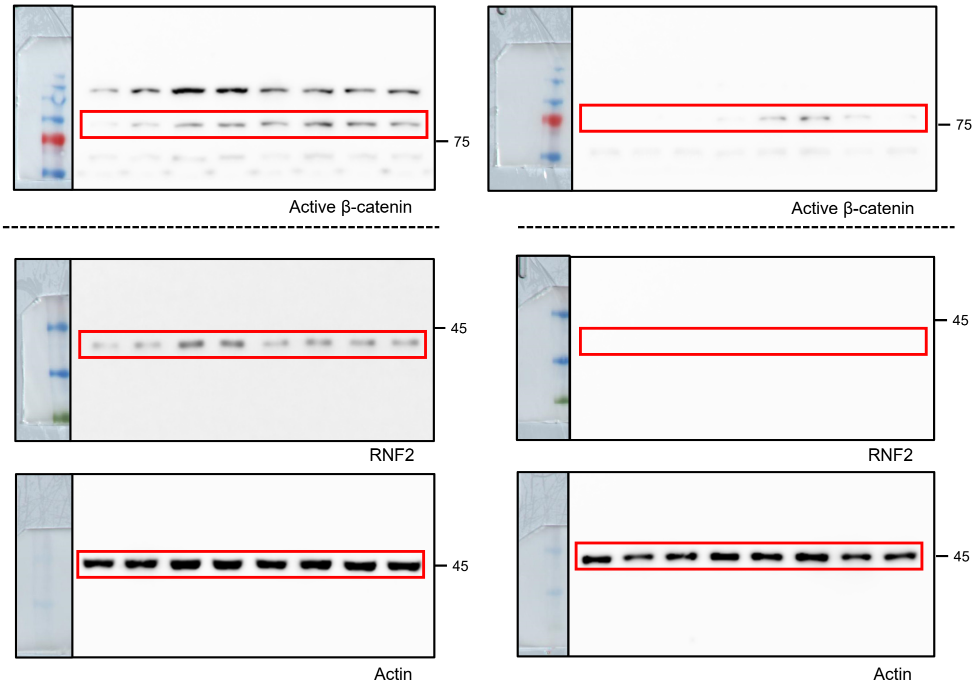

Figure 6b

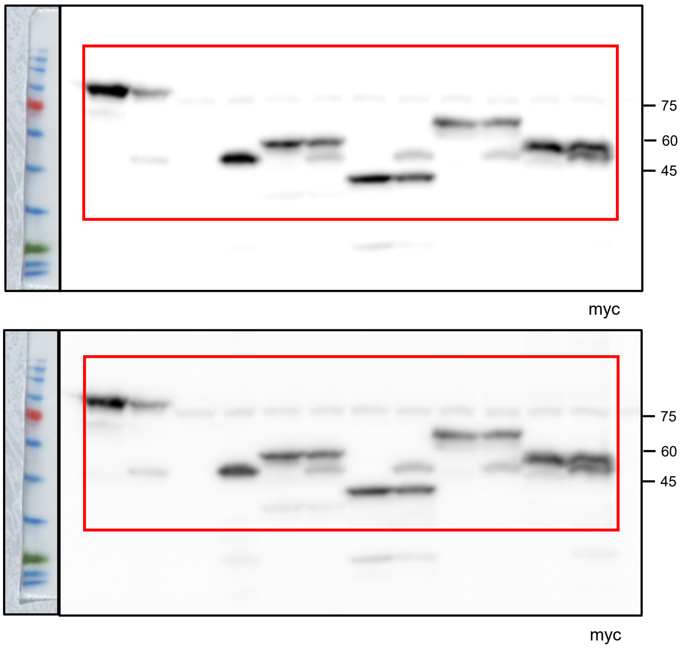

Figure S11

Figure 3b

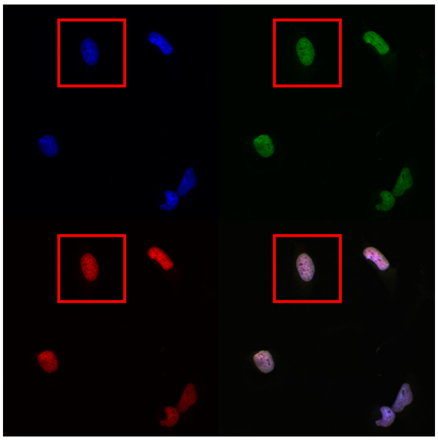

Figure 4c

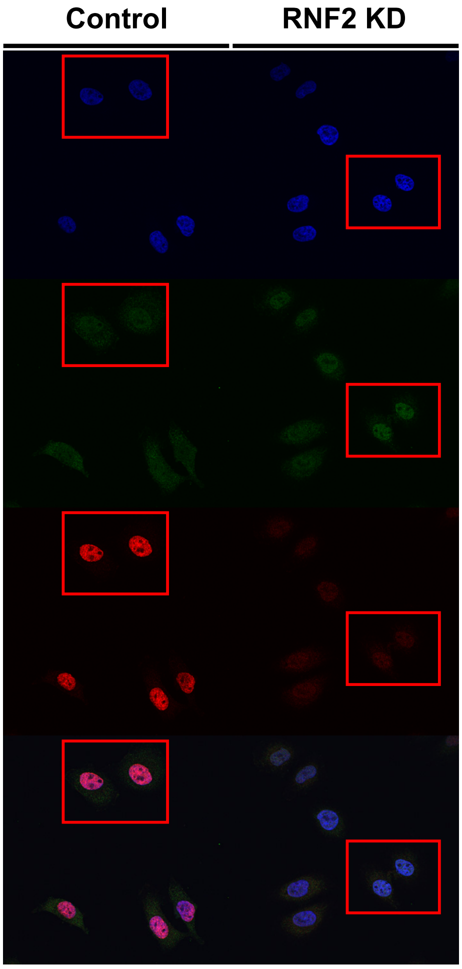

Figure 4d

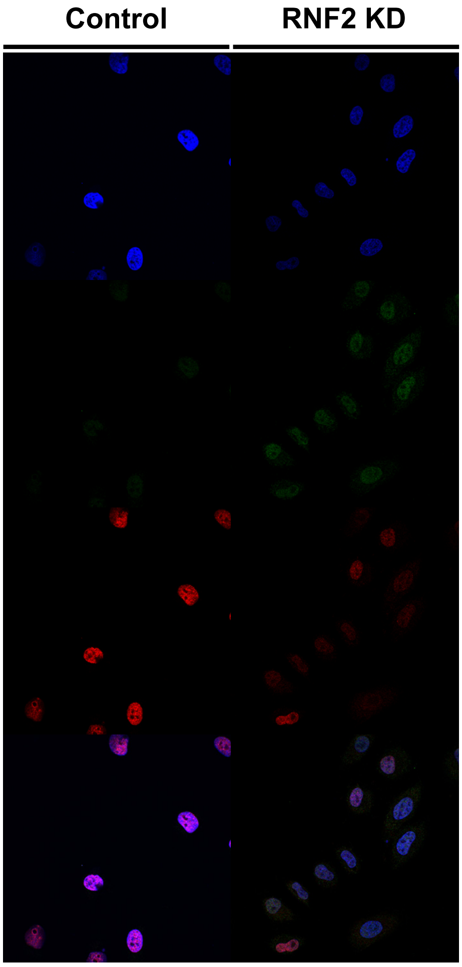

Figure S12

Figure 5d

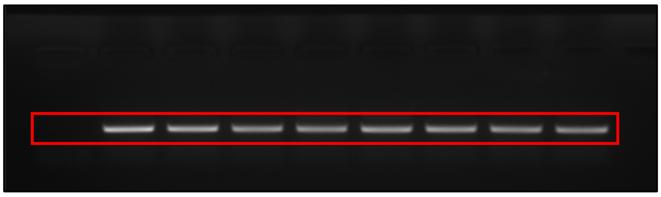

c-myc

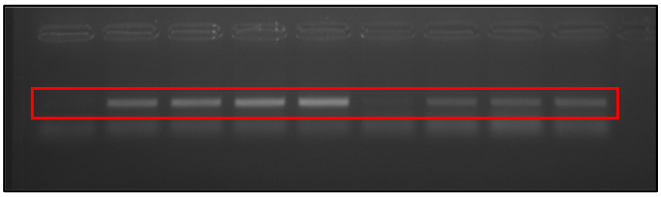

Axin2

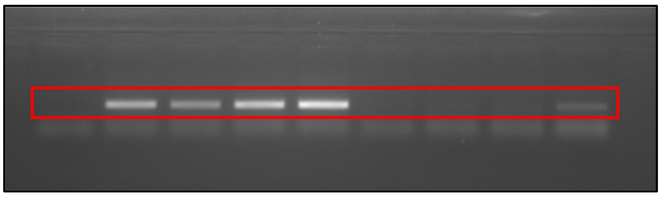

TCF1

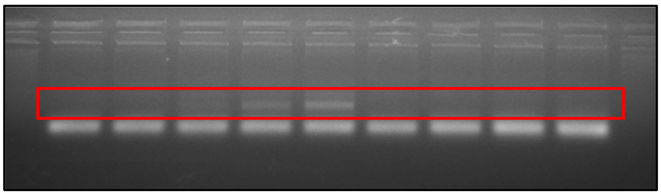

LEF1

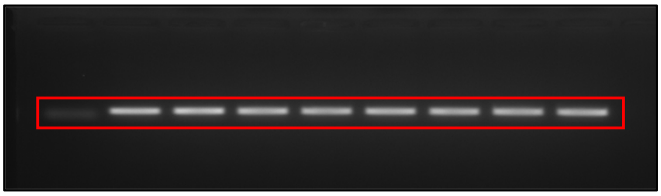

GAPDH

Figure S13

Figure S1e

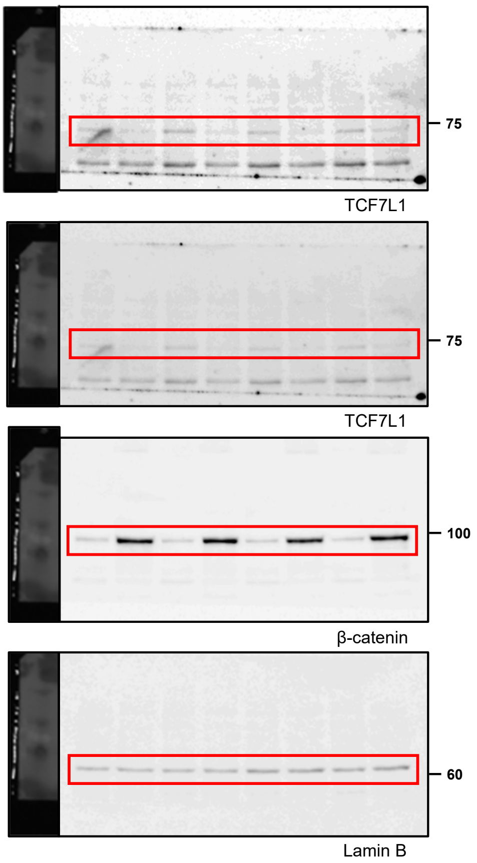

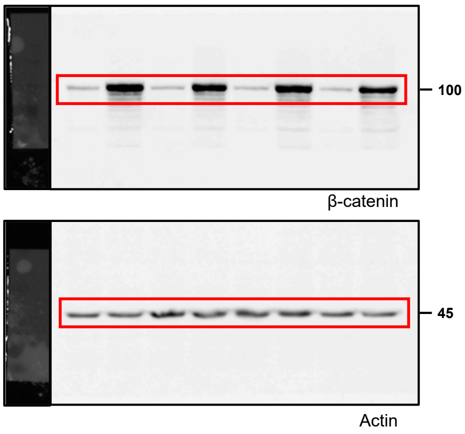

Figure S2a

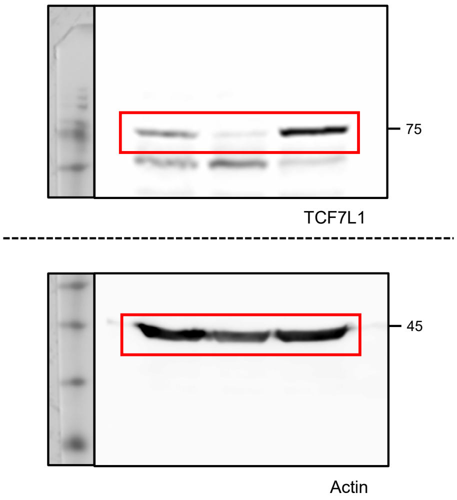

Figure S2c

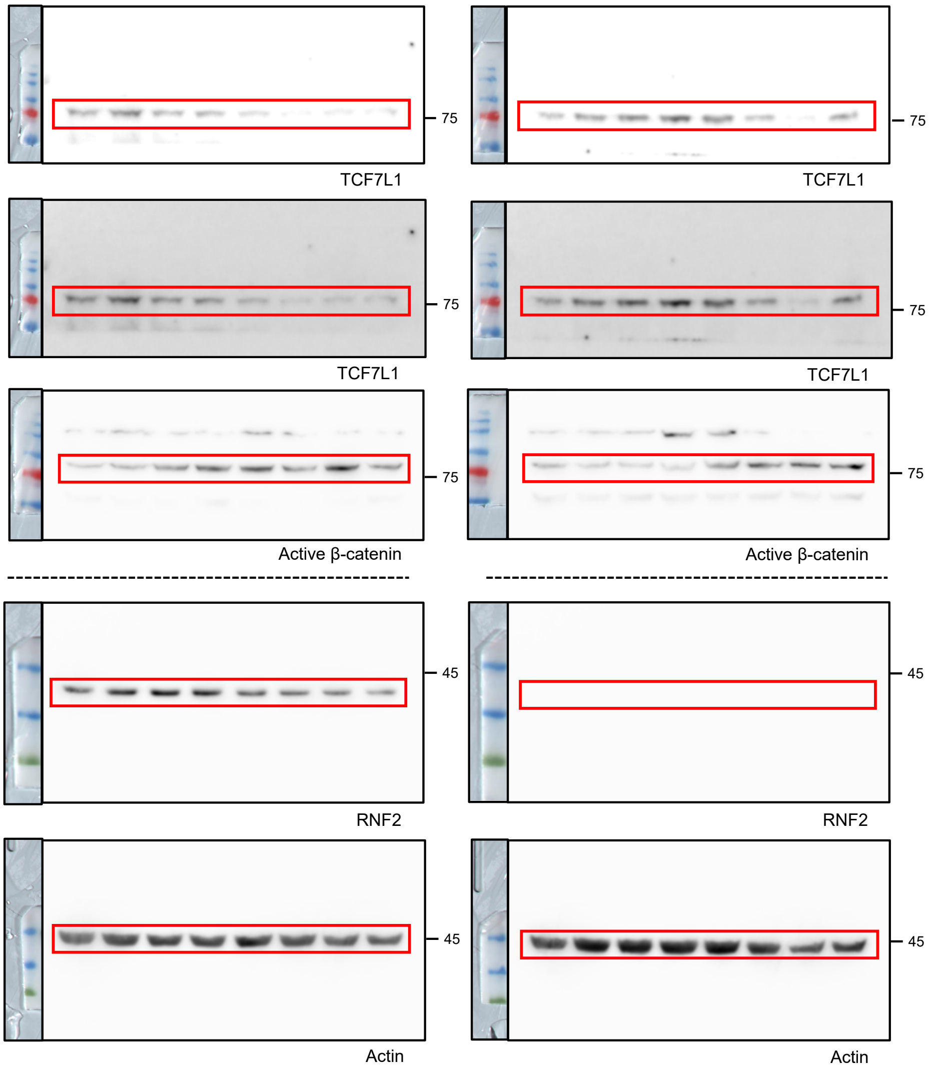

Figure S3a

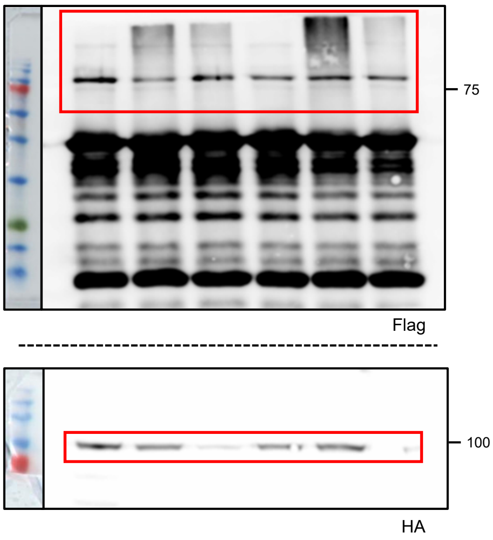

Figure S4b

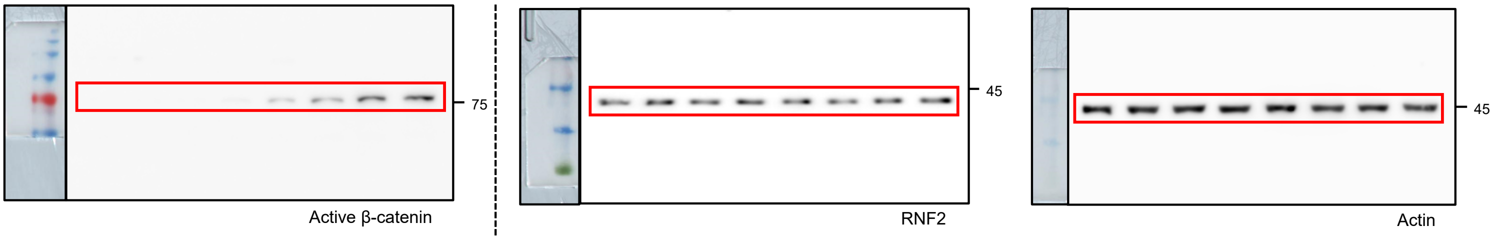

Figure S14

Figure S1b

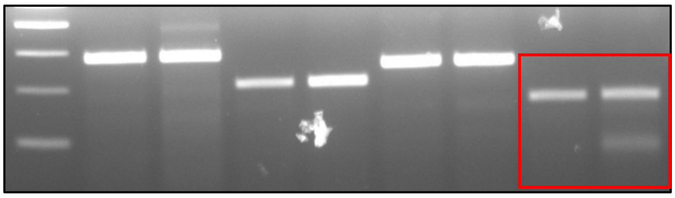

USP7 partial sequence

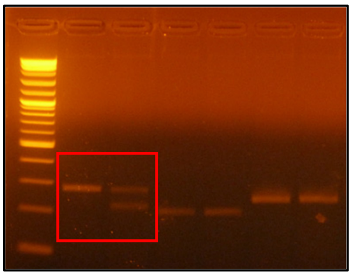

STUB1 partial sequence

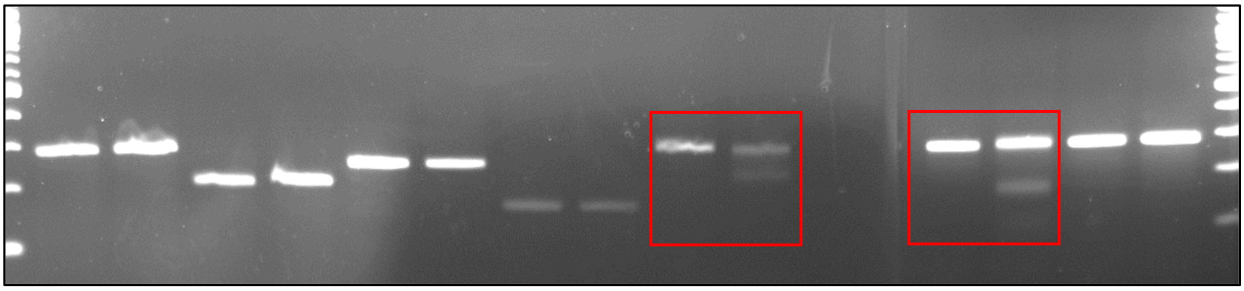

RNF2 partial sequence RNF169 partial sequence

Figure S4c

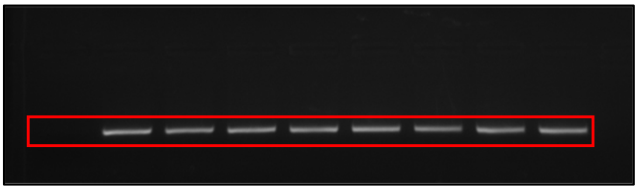

c-myc

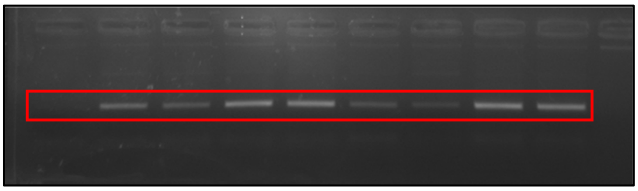

Axin2

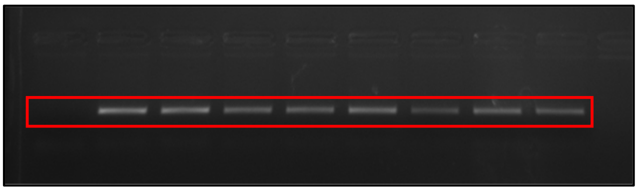

TCF1

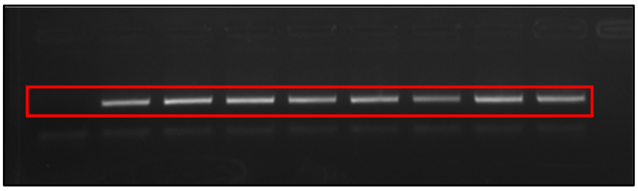

LEF1

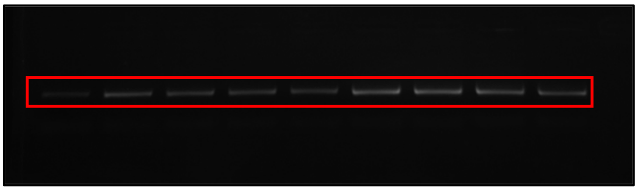

RNF2

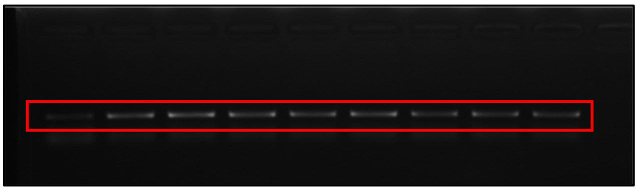

GAPDH

Figure S5a

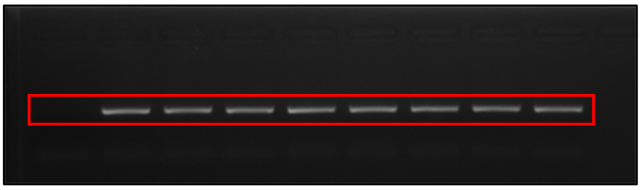

c-myc

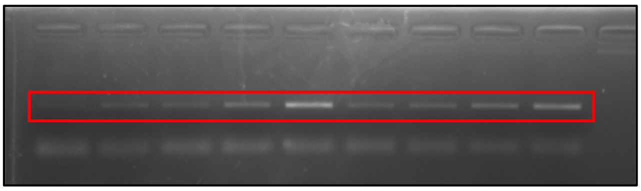

Axin2

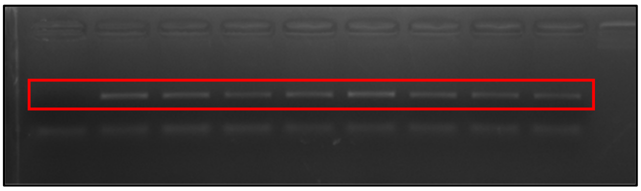

TCF1

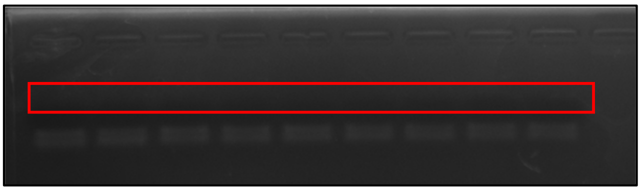

LEF1

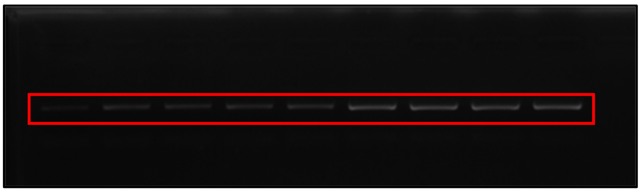

RNF2

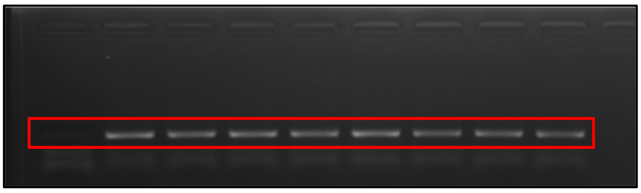

GAPDH

Figure S15

Figure S6a

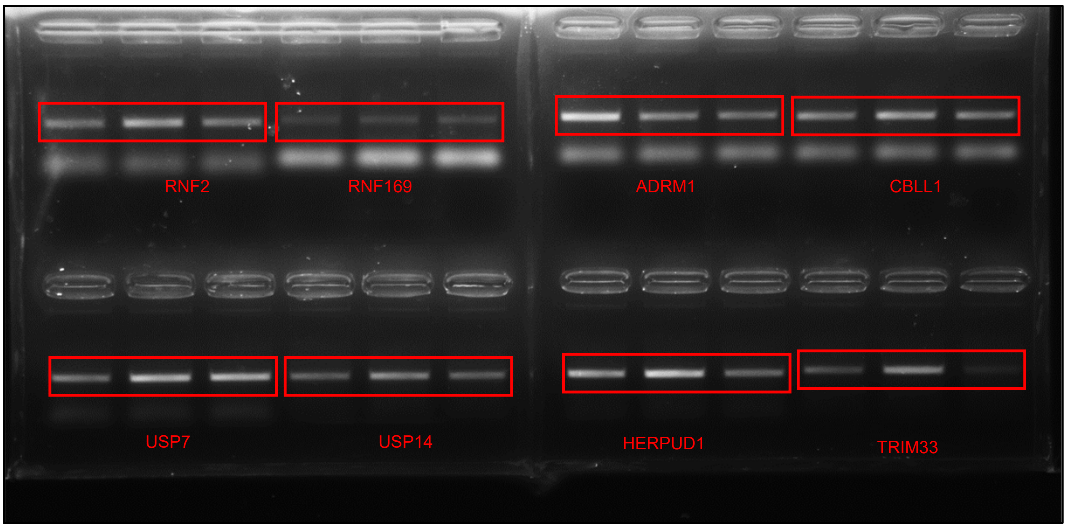

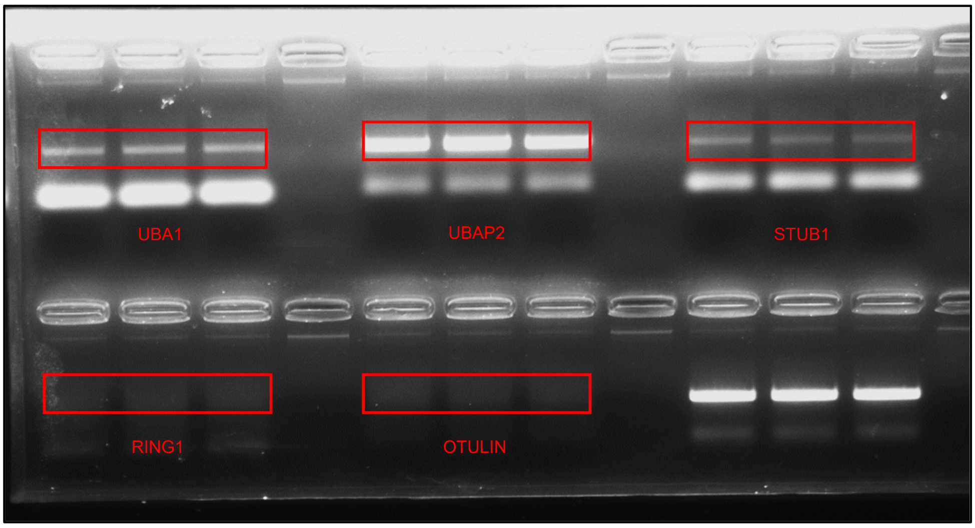

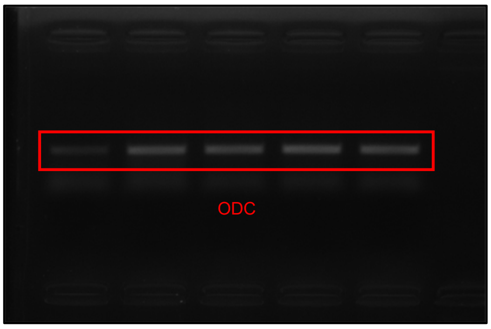

Figure S6b

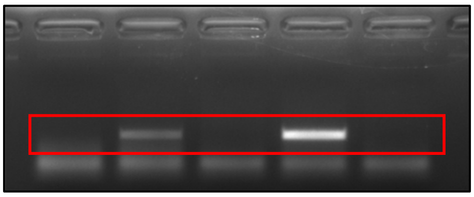

Chd

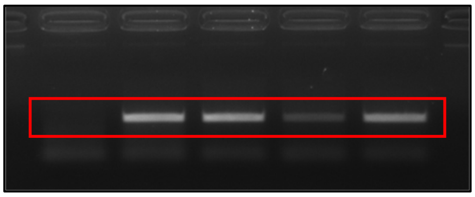

Msx1

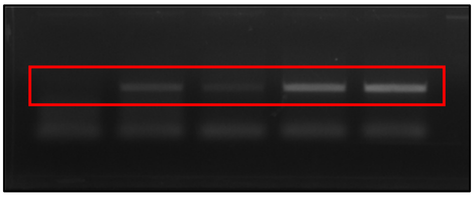

Xbra

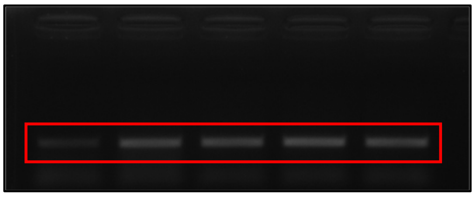

ODC

Figure S1. Validation of candidates from mass spectrometry result
a. qRT-PCR was performed to validate knockdown efficiency of stable knockdown HeLa cells. The target mRNA levels were normalized by GAPDH. b. Mutation detection assay was performed using sgRNA based stable knockdown cell lines. Double band demonstrated the mutation of target sequence is occurred. c. TOPflash luciferase assay was performed using control HeLa cells. Cells were transfected with the indicated plasmids (50 ng TK-Renilla reporter; 200 ng TOPflash; 0.5 pCS2+TCF7L1; 1 ng pt-b-catenin) and then treated with 50 mM LiCl conditioned media for 16 hours. N = 3 independent experiments. d. TOPflash luciferase assay was performed using control HeLa cells and stable knock-down cell lines. Cells were transfected with the indicated plasmids (50 ng TK-Renilla reporter; 200 ng TOPflash) and then treated with 50 mM NaCl and LiCl conditioned media for 16 hours. n=3 independent experiments. e. Western blot analysis was performed using stable knockdown HeLa cells. Cells were treated with 50 mM LiCl for 16 hours. The cytosolic fraction was lysed with CB buffer, while the nuclear fraction was lysed with RIPA buffer following the initial lysis with CB buffer.

Figure S2. Loss of RNF2 in 4T1 cell line also showed increased TCF7L1 expression
a. Western blot assay was performed using HeLa, HEK293T and 4T1. Endogenous TCF7L1 levels were determined by anti-TCF7L1. b. Western blot analysis of control 4T1 and shRNA-based RNF2 knockdown stable 4T1 cells. Endogenous TCF7L1 and RNF2 levels were determined using anti-TCF7L1 and anti-RNF2 antibodies, respectively. Quantification was performed using ImageJ software. c. Western blot analysis of control 4T1 cells and RNF2 knockdown stable 4T1 cells treated with 50 mM LiCl conditioned media for varying durations (30 min, 1 hr, 2 hrs, 4 hrs, 8 hrs, 16 hrs, and 24 hrs).

Figure S3. Both RNF2 overexpression and LiCl treatment accelerates TCF7L1 degradation
a. In vitro ubiquitination assay was performed using control HeLa cells. TCF7L1-HA (6 µg), myc-RNF2 plasmids (6 µg) and Flag-Ub (2 µg) were introduced into cells. Cells were treated with 50 mM LiCl conditioned media for 16 hours followed by treatment with 10 µM MG132 for 12 hours, prior to harvesting. The cells were lysed with IP buffer and subjected to immunoprecipitation with the HA antibody. Ubiquitination levels were determined by anti-OctA.

Figure S4. Supplement data using HEK293T cells
a. HEK293T cells were fixed and stained with anti-TCF7L1 (green) and anti-RNF2 (red) antibodies. Scale bar represents 10 µm. b. Western blot analysis of control HEK293T cells treated with 50mM LiCl conditioned media for varying durations (30min, 1 hr, 2hrs, 4 hrs, 8 hrs, 16 hrs and 24 hrs). c. RT-PCR analysis of total RNAs extracted from control HEK293T cells and RNF2-myc introduced HEK293T cells after treatment with LiCl (50 mM) conditioned media for 4, 8, and 12 hours. Samples were subjected to cDNA synthesis and RT-PCR analysis.

Figure S5. Wnt signaling activity is not affected by RNF2 overexpression
a. RT-PCR analysis of total RNAs extracted from control HeLa cells and myc-RNF2 introduced HeLa cells after treatment with LiCl (50 mM) conditioned media for 4, 8, and 12 hours. Samples were subjected to cDNA synthesis and RT-PCR analysis. b. Quantification of RNF2 mRNA level using ImageJ from HeLa control cells and myc-RNF2 (6 µg) plasmid introduced HeLa cells after 50mM LiCl treatment for 4, 8, 12 hours. The intensity of RNF2 was normalized by that of GAPDH. N = 3 independent experiments.

Figure S6. TCF7L1 and RNF2 expression during *Xenopis laevis* development
a. RT-PCR analysis was performed using *Xenopus laevis* stage 11 embryo. Animal cap(AC), Dorsal marginal zone (DMZ), Ventral marginal zone(VMZ) tissues were surgically dissected. The tissues were subjected to cDNA synthesis and RT-PCR analysis. b. RT-PCR analysis was performed using *Xenopus laevis* stage 11 embryo. Organizer, mesoderm and neural crest was determined by Chordin(Chd), Xbra, Msx1, respectively. c. RNA-seq data of TCF7L1 and RNF2 in Session et al. (2016) d. Tissue profile of TCF7L1 and RNF2 in Session et al. (2016) e. In situ hybridization was performed. Spatio-temporal expression pattern of TCF7L1 and RNF2 during *Xenopus* development. f. RNF2 overexpression was performed through RNF2 microinjection was performed. Capped RNF2 mRNA was injected in dorsal-animal side of 4-cell stage embryo.

Figure S7-S10. Full-sized images of western blot data from the main figures.

Figure S11. Original images of immunofluorescence data from the main figures.

Figure S12. Full-sized images of RT-PCR data from the main figures.

Figure S13. Full-sized images of western blot data from the supplement figures.

Figure S14-S15. Full-sized images of RT-PCR data from the supplement figures.
