# Supplementary material for: RNF2 regulates Wnt/ß-catenin signaling via TCF7L1 destabilization
Source: Sci Rep. 2023 Nov 13;13:19750. doi: 10.1038/s41598-023-47111-x (PMC10643375; doi:10.1038/s41598-023-47111-x)
Supplement: Supplementary file 2 — Supplementary Tables. [file 41598_2023_47111_MOESM2_ESM.pdf]

Supplementary Information

10 Tables

RNF2 regulates Wnt/ß-catenin signaling via TCF7L1 destabilization

Youngmu Koo[1], Wonhee Han[2], Byeong-Rak Keum[1], Leila Lutz[1], Sung Ho Yun[3], Gun-Hwa Kim[4], Jin-Kwan Han[1,*]

1. Department of Life Sciences, Pohang University of Science and Technology, 77 Cheongam-Ro, Nam-Gu, Pohang, Gyeongbuk, 37673, Korea.

2. F. M. Kirby Neurobiology Center, Boston Children's Hospital, Department of Neurology, Harvard Medical School, Boston, MA 02115, USA.

3. Center for Research Equipment, Korea Basic Science Institute, Cheongju 28119, Republic of Korea

4. Research Center for Bioconvergence Analysis, Korea Basic Science Institute, Cheongju 28119, Republic of Korea

* Corresponding author: jkh@postech.ac.kr

Table 1

Genes	Full name	Role	Rank
**TRIM33**	E3 ubiquitin-protein ligase TRIM33	E3 ligase	854
**RNF2**	E3 ubiquitin-protein ligase RING2	E3 ligase	39
**RNF169**	E3 ubiquitin-protein ligase RNF169	E3 ligase	203
**CBLL1**	E3 ubiquitin-protein ligase Hakai	E3 ligase	980
**RING1**	Isoform 2 of E3 ubiquitin-protein ligase RING1	E3 ligase	29
**STUB1**	E3 ubiquitin-protein ligase CHIP	E3 ligase	1014
**USP7**	Ubiquitin carboxyl-terminal hydrolase 7	DUB	134
**USP14**	Isoform 3 of Ubiquitin carboxyl-terminal hydrolase 14	DUB	1126
**OTULIN**	Ubiquitin thioesterase otulin	DUB	1390
**UBA1**	Ubiquitin-like modifier-activating enzyme 1	E1 enzyme	585
**HERPUD1**	Homocysteine-responsive endoplasmic reticulum-resident ubiquitin-like domain member 1 protein	ER protein with Ubiquitin like domain	16
**UBAP2**	Ubiquitin-associated protein 2	Ubiquitin associated protein2	509
**UBAP2L**	Isoform 5 of Ubiquitin-associated protein 2-like	Ubiquitin associated protein2 Like	1459
**URM1**	Ubiquitin-related modifier 1	Ubiquitin like protein	1356
**ADRM1**	Proteasomal ubiquitin receptor ADRM1	Ubiquitin receptor	269

Table 2

Candidate	Role	Rank (1470)
RING1	E3 ligase	29
RNF2	E3 ligase	39
RNF169	E3 ligase	203
TRIM33	E3 ligase	854
CBLL1	E3 ligase	980
STUB1	E3 ligase	1014

Table 3

Genes	Target sequence	Primer sequences (5’→3')
STUB1	GAAGAGGAAGAAGCGAGACAT	CCGGGAAGAGGAAGAAGCGAGACATCTCGAGATGTCTCGCTTCTTCCTCTTCTTTTTG
		AATTCAAAAAGAAGAGGAAGAAGCGAGACATCTCGAGATGTCTCGCTTCTTCCTCTTC
URM1	GCTCCTGTTTGACGGTATTAA	CCGGGCTCCTGTTTGACGGTATTAACTCGAGTTAATACCGTCAAACAGGAGCTTTTTG
		AATTCAAAAAGCTCCTGTTTGACGGTATTAACTCGAGTTAATACCGTCAAACAGGAGC
UBAP2L	GCCAATACTGATGATAACTAT	CCGGGCCAATACTGATGATAACTATCTCGAGATAGTTATCATCAGTATTGGCTTTTTG
		AATTCAAAAAGCCAATACTGATGATAACTATCTCGAGATAGTTATCATCAGTATTGGC
UBAP2	CGTGGAAACAACAACCGGAAA	CCGGCGTGGAAACAACAACCGGAAACTCGAGTTTCCGGTTGTTGTTTCCACGTTTTTG
		AATTCAAAAACGTGGAAACAACAACCGGAAACTCGAGTTTCCGGTTGTTGTTTCCACG
RNF169	ATCAACTCCACGCAACCTAAA	CCGGATCAACTCCACGCAACCTAAACTCGAGTTTAGGTTGCGTGGAGTTGATTTTTTG
		AATTCAAAAAATCAACTCCACGCAACCTAAACTCGAGTTTAGGTTGCGTGGAGTTGAT
ADRM1	CAGACGGACGACTCGCTTATT	CCGGCAGACGGACGACTCGCTTATTCTCGAGAATAAGCGAGTCGTCCGTCTGTTTTTG
		AATTCAAAAACAGACGGACGACTCGCTTATTCTCGAGAATAAGCGAGTCGTCCGTCTG
UBA1	CCACTGCCTTCTACCTTGTTT	CCGGCCACTGCCTTCTACCTTGTTTCTCGAGAAACAAGGTAGAAGGCAGTGGTTTTTG
		AATTCAAAAACCACTGCCTTCTACCTTGTTTCTCGAGAAACAAGGTAGAAGGCAGTGG
RNF2	GCCAGGATCAACAAGCACAAT	CCGGGCCAGGATCAACAAGCACAATCTCGAGATTGTGCTTGTTGATCCTGGCTTTTTG
		AATTCAAAAAGCCAGGATCAACAAGCACAATCTCGAGATTGTGCTTGTTGATCCTGGC
RING1	CTGGAGCTGGTGAATGAGAAA	CCGGCTGGAGCTGGTGAATGAGAAACTCGAGTTTCTCATTCACCAGCTCCAGTTTTTG
		AATTCAAAAACTGGAGCTGGTGAATGAGAAACTCGAGTTTCTCATTCACCAGCTCCAG
HERPUD1	CCTCCTCCTGACGTTGTAAAT	CCGGCCTCCTCCTGACGTTGTAAATCTCGAGATTTACAACGTCAGGAGGAGGTTTTTG
		AATTCAAAAACCTCCTCCTGACGTTGTAAATCTCGAGATTTACAACGTCAGGAGGAGG
OTULIN	CCCTTTAGTAGTAACGGGTTT	CCGGCCCTTTAGTAGTAACGGGTTTCTCGAGAAACCCGTTACTACTAAAGGGTTTTTG
		AATTCAAAAACCCTTTAGTAGTAACGGGTTTCTCGAGAAACCCGTTACTACTAAAGGG
USP14	CCCAAGATTCAGCAGTCAGAT	CCGGCCCAAGATTCAGCAGTCAGATCTCGAGATCTGACTGCTGAATCTTGGGTTTTTG
		AATTCAAAAACCCAAGATTCAGCAGTCAGATCTCGAGATCTGACTGCTGAATCTTGGG
CBLL1	TCATATCAACCATCGCCATAT	CCGGTCATATCAACCATCGCCATATCTCGAGATATGGCGATGGTTGATATGATTTTTG
		AATTCAAAAATCATATCAACCATCGCCATATCTCGAGATATGGCGATGGTTGATATGA
TRIM33	TACTTTCCAGTTGCGTCATAT	CCGGTACTTTCCAGTTGCGTCATATCTCGAGATATGACGCAACTGGAAAGTATTTTTG
		AATTCAAAAATACTTTCCAGTTGCGTCATATCTCGAGATATGACGCAACTGGAAAGTA
USP7	CCTGGATTTGTGGTTACGTTA	CCGGCCTGGATTTGTGGTTACGTTACTCGAGTAACGTAACCACAAATCCAGGTTTTTG
		AATTCAAAAACCTGGATTTGTGGTTACGTTACTCGAGTAACGTAACCACAAATCCAGG

Table 4

Genes	Primer sequences (5’→3')
UBAP2L	TGAGCCACAGTGAGGAGATTCC
	AGATTCGCCTCACTCTCCACAC
OTULIN	GACAGCTTCTGAGGAACCACCT
	TCCGTGTTGTACTTGGAGAGCC
URM1	CATCGAGTCACTTTGCCTGGAC
	ACGCTGTCTCCCTGGATGAACA
USP14	GGGAAATGGCTTCAGCGCAGTA
	CACCTTTCTCGGCAAACTGTGG
HERPUD1	CCAATGTCTCAGGGACTTGCTTC
	CGATTAGAACCAGCAGGCTCCT
CBLL1	AACAGGATGCCTGCAAAGGCTC
	GGTGTCCAGGAAATCTTCGCTG
ADRM1	AAGCGGAAAGGGCTGGTGTACA
	CCGCTTGAACTCACAGTCGTCA
UBA1	TCCTCACAGAGGACAAGTGCCT
	CTTGAGCAGCTCACAGCCAATG
RNF2	CAGTCACAGCATTGAGGAAGGAC
	GCTTCCTGATTGCTATGTGTGGA

Table 5

Genes	Target sequence	Primer sequences (5’→3')
RNF2	TGAGTTACAACGAACACCTC	CACCGTGAGTTACAACGAACACCTC
		AAACGAGGTGTTCGTTGTAACTCAC
RNF169	GAGTACTCGGGCCTCTTCCG	CACCGGAGTACTCGGGCCTCTTCCG
		AAACCGGAAGAGGCCCGAGTACTCC
STUB1	GAATCGCGAAGAAGAAGCGC	CACCGGAATCGCGAAGAAGAAGCGC
		AAACGCGCTTCTTCTTCGCGATTCC
TRIM33	GTGTTGTGTACCGATACTAC	CACCG CATCATAGCCATGTGTTGTG
		AAACCACAACACATGGCTATGATGC
USP7	AGATGTATGATCCCAAAACG	CACCG AGATGTATGATCCCAAAACG
		AAACCGTTTTGGGATCATACATCTC
RING1	TAAGATCTATCCTAGCCGGG	CACCG TAAGATCTATCCTAGCCGGG
		AAACCCCGGCTAGGATAGATCTTAC

Table 6

Genes	Primer sequences (5’→3')
RNF2	AAACGGAACTCAACCATTAAGC
	TTATTCACCACTGTTCACCCAG
RNF169	AGGTCTGAGGAGAACCCTCTTT
	CAACAGCGAAGGTCCAGAAG
STUB1	GCTGGAGATGGAGAGCTATGAT
	ATGAGCCTGGAGAGGTAGGAGT
TRIM33	GCCCACTTCTTTGTTTCTTCTG
	GCATTTGCCATAGTTGCTGTAG
USP7	TTGTGCTGAGTGTTTGGATCTT
	GAAGGTAAGTGCACGAAGGACT
RING1	CAGGAACAAGGAGTGTCCTACC
	CCATCAGTTCTGCTTCTCTCCT

Table 7

Antibody	Catalog	Company
β-Actin(C4)	sc-47778	Santa cruz
TCF3(E-2)	sc-166411	Santa cruz
TCF4(H-7)	sc-271287	Santa cruz
c-myc(9E10)	sc-40	Santa cruz
HA-tag(F-7)	sc-7392	Santa cruz
β-Catenin(H-102)	sc-7199	Santa cruz
Lamin B1(B-10)	sc-374015	Santa cruz
OctA(H-5)	sc-166355	Santa cruz
Active-β-Catenin(8E7)	05-665	Sigma-Aldrich
K48-linkage Specific Polyubiquitin	#4289	Cell Signaling Technology
RING1B (D22F2)	#5694	Cell Signaling Technology

Table 8

Genes	Primer sequences (5'→3')
RNF2	GTCGTGATGAGTATGAAGCTC
	TTGTTACTAGGGCCTGCTTC
TCF7L1	AGAACCAGAGCAGCAGCTC
	TTCATCTGCAGGTAGGTGCG
c-myc	AGAAGCTGGCCTCCTACCA
	GTCGAGGAGAGCAGAGAATC
Axin2	GGCATCAAGAAGCAGCAGAT
	TTTGCACTTGAAGTCGGCAC
TCF1	CCGTCTACTCCGCCTTCAAT
	AGGTCAGGGAGTAGAAGCCA
LEF1	ACGGAAAGCATCCAGATGGA
	ATGTGTGACGGGTGTGATCC
GAPDH	GTCTCCTCTGACTTCAACAGCG
	ACCACCCTGTTGCTGTAGCCAA

Table 9

Genes	Primer sequences (5'→3')
USP7	CATTGACTATGTAGCAGTGGAG
	TTTGCCTTCTCCTTTAGGATTCAA
RNF169	CCAACTAGTGAATATGCAAAATGG
	CTAATTTGCGCCGGCCGTG
RNF2	GGCGCAAGCAGTGCAGAC
	CATCAAAGTTCGGATCTGGCC
USP14	TGCCCCTCTACTCAGTGAAC
	GTGTTACCGAGGTTTGTCAGG
ADRM1	GTCATCTGGAGCGCTGTTTC
	TCAGTCTTGGGCTCCTGCAT
TRIM33	GGCGGATAACAAAGGAGGAG
	GCTTGGGCTCAGTGTCCC
HERPUD1	ATACGGAGCCCTAGCCTTAG
	CTAATGCCATCAGCCTCAGG
CBLL1	GATAATGATCTACAAGGCACTAAC
	AGGCAGGCCACATTTGTCAC
RING1	CGCCAGTCTCGCGTGATG
	AATCTGTGGAGACATTCCTTGG
UBA1	GTCCAGCTCTCCGCTGTC
	GAAAGATCAGTCCACTCCGTG
UBAP2	ATGATGACTTCGGTGGGAAATG
	GGAACTCTCTTTGCCAATGCTT
STUB1	GAAAGGAAAGGAGGAGGGTG
	TCTCTAGTTCCATCTGACACTG
OTULIN1	CACCAGAGTAGCTAAAATGAAAATC
	GCTGAGAGCACTCTATGGAAC

Table 10

Genes	Primer sequences (5'→3')
TCF7L1	ATCTTGGAGGGCATTACTTGCC
	TGGGACTAATGTTCCCAGAGGA
RNF2	CAACGAAAGAGTGCTTGCATCG
	TGTATCGAGTCTGTGCACTGTC

Table 1. Candidate genes from mass spectrometry result

Table 2. Six E3 ligase candidate genes from the mass spectrometry data

Table 3. Primers used for shRNA

Table 4. Primers used for knockdown validation

Table 5. Primers used for making gRNA included PX459 vector

Table 6. Primers used for mutation detection

Table 7. Antibody information used for western blot

Table 8-9. Primers used for RT-PCR

Table 10. Primers used for cloning probe
